# The First Contact of Human Dendritic Cells With *Trypanosoma cruzi* Reveals Response to Virus as an Unexplored Central Pathway

**DOI:** 10.3389/fimmu.2021.638020

**Published:** 2021-04-09

**Authors:** Natalia Gil-Jaramillo, Amanda Pereira Rocha, Tainá Raiol, Flávia Nader Motta, Cecília Favali, Marcelo M. Brigido, Izabela M. D. Bastos, Jaime M. Santana

**Affiliations:** ^1^ Laboratório de Interação Patógeno-Hospedeiro, Instituto de Ciências Biológicas, Universidade de Brasília, Brasília, Brazil; ^2^ Fiocruz Brasília–Gerência Regional de Brasília (GEREB), Fundação Oswaldo Cruz (Fiocruz), Brasília, Brazil; ^3^ Faculdade de Ceilândia, Universidade de Brasília, Brasília, Brazil; ^4^ Laboratório de Imunologia Celular, Instituto de Ciências Biológicas, Universidade de Brasília, Brasília, Brazil; ^5^ Laboratório de Imunologia Molecular, Instituto de Ciências Biológicas, Universidade de Brasília, Brasília, Brazil

**Keywords:** Chagas disease, inflammasome, metacyclic trypomastigotes, transcriptome, virus infection

## Abstract

Chagas disease is a debilitating and neglected disease caused by the protozoan *Trypanosoma cruzi*. Soon after infection, interactions among *T. cruzi* and host innate immunity cells can drive/contribute to disease outcome. Dendritic cells (DCs), present in all tissues, are one of the first immune cells to interact with *Trypanosoma cruzi* metacyclic trypomastigotes. Elucidating the immunological events triggered immediately after parasite-human DCs encounter may aid in understanding the role of DCs in the establishment of infection and in the course of the disease. Therefore, we performed a transcriptomic analysis of a 12 h interaction between *T. cruzi* and MoDCs (monocyte-derived DCs) from three human donors. Enrichment analyses of the 468 differentially expressed genes (DEGs) revealed viral infection response as the most regulated pathway. Additionally, exogenous antigen processing and presentation through MHC-I, chemokine signaling, lymphocyte co-stimulation, metallothioneins, and inflammasome activation were found up-regulated. Notable, we were able to identify the increased gene expression of alternative inflammasome sensors such as AIM2, IFI16, and RIG-I for the first time in a *T. cruzi* infection. Both transcript and protein expression levels suggest proinflammatory cytokine production during early *T. cruzi*-DCs contact. Our transcriptome data unveil antiviral pathways as an unexplored process during *T. cruzi*-DC initial interaction, disclosing a new panorama for the study of Chagas disease outcomes.

## Introduction

Chagas disease, caused by *Trypanosoma cruzi*, is an endemic parasitic disease that affects approximately seven million people in Latin America, with over 65 million estimated to be at risk of contracting the disease (1, 2). The disease has turned into a global health concern, mainly because of migratory flow and lack of treatment or vaccine (3–6). Individuals who are infected by the parasite initially develop an acute asymptomatic phase, with only 1% manifesting non-specific symptoms (7). After 20 to 30 years, however, approximately one-third of infected individuals will progress to the chronic phase, which is characterized by cardiomegaly and mega viscera, which can lead to death (8, 9). The outcome of the infection depends upon the combination of many complex processes, with accurate prediction regarding progression to the chronic phase remaining a challenge. Immunological events within the acute phase influence the development of either a protective or a pathogenic response in later stages of Chagas disease (10). Likewise, parasite genetic diversity and tissue tropism, inoculum, and transmission route, together with host genetic background, age, and nutritional conditions, may all modulate immunological and pathogenesis mechanisms (10, 11).

Chagas disease is a vector-borne disease, with the parasite transmitted by blood-sucking bugs from the Triatominae family (12). Its oral transmission is of increasing epidemiological importance, with frequent outbreaks of acute and, at times, lethal cases. In these episodes, *T. cruzi* is acquired through food or drink products contaminated with bugs and/or bug feces/urine (13, 14). During its complex life cycle, the parasite evolves through different forms, including epimastigotes, the insect replicative midgut forms; metacyclic trypomastigotes (MTs), the infective and non-replicative rectum insect forms; amastigotes, the intracellular vertebrate replicative forms; and bloodstream trypomastigotes, the infective and non-replicative vertebrate forms (12, 15). Natural insect-derived MTs are found in triatomine feces/urine and invade vertebrate cells through the insect bite site or mucosal tissues (13, 16). MTs are thus responsible for the parasite’s first interaction with the host cells.

MT has a variety of cellular membrane and secreted virulence factors that allow for mammalian cell invasion, host immune response evasion, and parasite intracellular survival (17). Once inside the host, the parasite may be recognized and able to infect phagocytic and/or non-phagocytic cells through a variety of host cell factors. Among the molecules on non-phagocytic cells, mucins, laminins, fibronectins, thrombospondins, heparan sulfate proteoglycans, and bradykinin B_2_ receptors have been reported to mediate parasite entrance (18–22). Otherwise, classic and non-classic pattern recognition receptors (PRRs) such as TLR2, TLR4, TLR9, NOD2, lectins, C5a, Slamf1, and LAMP-2 have shown their importance for *T. cruzi* internalization in monocytes, macrophages, and dendritic cells (DCs) (23–28).

*Ex vivo* studies in indeterminate patients have shown that the activation of Th1, Th2, Treg, and Th17 cells can lead to the control of parasitemia and tissue damage and the development of immunological memory against *T. cruzi* (29–31). However, an excessive and/or prolonged response of any of these types may contribute to the disease pathogenesis (32). Therefore, the signaling pathways unleashed by the host innate immune system immediately after sensing the infection will impact almost every aspect of the subsequent adaptative immune response (33, 34). In this sense, DCs perform a crucial role in controlling infection at the beginning of the acute phase and in coordinating innate and adaptive immune response (35).

Since they are present in all tissues, DCs show potential to be the first immune cells to interact with the pathogen (36). Nevertheless, the DC molecular signature, subtypes, and maturation are needed to provide a precise understanding of these cell functions (37), especially under *T. cruzi* infection. Recently, it was demonstrated that several subtypes of murine DCs, examined after infection by *T. cruzi*, showed a lower expression of MHC class II when compared to the steady-state (35). A lower expression of MHC class I and II was also found when human monocyte-derived DCs (MoDCs) interacted with the parasite (38–41). T cell co-stimulatory molecules CD80, CD86, CD83, and CD40 showed a reduced expression on murine DC surface after infection with different *T. cruzi* strains. On the other hand, the expression of PD-L1, a molecule that suppresses the proliferation of antigen-specific T-cells, was decreased (42–44). Regarding cytokine production, in most cases, human and murine models showed an increased production of anti-inflammatory cytokines IL-10, TGF-β, and IL-4, responsible for host susceptibility to parasite infection, and a decreased production of IL-12 and TNF-α, important protective molecules against the parasite (28, 38, 39, 42–44). Therefore, *T. cruzi* seems to have the capacity of downregulating the expression of MHC class I and II molecules, co-stimulatory molecules, and proinflammatory cytokines, modulating host DCs that may help to modulate the adaptive immune response.

Studies on *T. cruzi*-DC interactions and their importance in the establishment of infection are scarce. In addition, the majority of published investigations are restricted to the murine model, hindering the capability to elucidate the first immunological events triggered immediately after parasite-human DC interaction (45). Moreover, data from murine Chagas disease immune response may not be extrapolated to human infection, which highlights the necessity of comprehending *T. cruzi*-DC interactions using human leukocytes. To address these goals, we carried out a comparative RNA-sequencing-based transcriptome analysis of human MoDCs after exposure to MTs *vs.* non-infected MoDCs. Analyses of differentially expressed gene (DEG) profiles revealed that *T. cruzi* is able to elicit a virus-related response in MoDCs. These discoveries open up new perspectives for the pathogenesis of this disease, with better-elucidated viral infection mechanisms expanding understanding of Chagas disease.

## Materials and Methods

### Parasite

*T. cruzi* CL Brener strain epimastigotes were cultivated in liver infusion tryptose (LIT) medium (46), pH 7.0, supplemented with 5% heat-inactivated fetal bovine serum (FBS) and 0.1 mg/ml gentamicin at 28°C. For metacyclogenesis, epimastigotes in final stationary phase were cultivated in triatomine artificial urine (TAU) medium for 2 h at 28°C as previously described (47). After this period, TAU was replaced by TAU3AAG medium (TAU medium added with 10 mM L-proline, 10 mM glutamic acid, 2 mM aspartic acid, and 10 mM glucose) and the parasites were cultivated for 6 days at 28°C. Parasites were harvested and incubated in 100% active FBS (non-heat inactivated) for 24 h to assure residual epimastigote lysis (48). Epimastigote cellular debris was decanted twice for 2 h and the resulting swimming MTs were washed thrice in PBS. Clean MTs were resuspended in RPMI 1640. The differentiated parasites (MTs) were quantified using instant prov stain (NewProv) to visualize the nucleus and kinetoplast localization.

### Donors

Donors were composed of three male and three female individuals between 22 and 33 years old ($\bar{x}$ = 28.3 ± 2.9) which were not previously infected with *T. cruzi*, as verified by PCR (**Figure S1**) (49). Infection and vaccination data from each donor were collected in **Table S1**. The study protocol was reviewed and approved by the Research Ethics Committee from the Medicine Faculty (Comitê de Ética em Pesquisa da Faculdade de Medicina)–The University of Brasília (CAAE: 54822616.7.0000.5558). Written informed consent for the work was collected from all subjects.

### Human MoDCs

Blood was collected from healthy donors and peripheric blood mononuclear cells (PBMCs) were obtained through density gradient centrifugation using Ficoll-Paque Plus (GE Healthcare). Monocytes were then purified from PBMCs using the CD14 microbead human kit (Miltenyi Biotec). Monocytes recovered from the positive fraction were cultivated at 37°C, 5% CO_2_ for 7 days in a concentration of 5 × 10^5^ cells/ml in RPMI 1640 medium (Gibco) supplemented with 10% heat-inactivated FBS (Gibco), 0.15 mg/ml gentamicin, 0.05 µg/ml of human GM-CSF (Peprotech), and 0.16 µg/ml of human IL-4 (Peprotech) (34, 50). Cytokine stimulus was maintained by replacing 10% of medium every 3-culture days. To evaluate activation/maturation in MoDCs, the three criteria of morphological changes considered were: size, presence of cytoplasmic prolongations, and non-spherical shape (36, 51, 52).

### MoDCs Infection

After DC differentiation, 5 × 10^5^ cells/ml were plated in 24-well plates at 37°C with 5% CO_2_ and infected with MTs in a 10:1 MOI for 12 h (final volume: 1.1 ml RPMI 1640). After this period, infected and control MoDCs were harvested, washed, and counted. To estimate the infection rate, 10^5^ MoDCs were collected and stained with instant prov stain (NEWPROV). A total of 300 cells were counted in double-blind format, identifying infected cells (infection rate) and amount of intracellular amastigotes per infected cell. An unpaired Student t-test was used to determine differences in infection rate, number of amastigotes per infected cell among donors, and activation between Infected and Control groups (n = 8).

### Cytokine Quantification

After infection, supernatant was collected and stored at −80°C until post-analysis. IL-1β, IL-8, IL-10, and TNF were measured using a cytometric bead array (CBA) Human Inflammatory Cytokine Cytometric Bead Array (CBA) - I Kit (BD) and analyzed by flow cytometry using a FACs Verse (BD Bioscience), following the manufacturer’s recommendations. Briefly, 50 µl of a solution containing the capture beads for each cytokine were mixed with 50 µl of cell culture supernatant and 50 µl of Human Inflammatory Cytokine PE Detection Reagent. After 3 h of incubation at room temperature, samples were washed and resuspended in the indicated buffer.

### RNA Extraction

Infected and control MoDCs were harvested and RNA extracted using Trizol reagent (Ambion), as suggested by the manufacturer’s recommendations. The final material was eluted in RNase free water and RNA integrity was assessed using bioanalyzer (Agilent Technologies). Samples with RINs below eight were excluded from the transcriptome analysis.

### RNA-seq

Two replicates from each donor were used to perform transcriptome sequencing at Novogen (http://www.novogen.com) according to their protocols. Briefly, oligo d(T) beads were employed to perform mRNA enrichment, which were then fragmented randomly and converted into cDNA. Poly(A) tails were added and enriched by PCR to generate a cDNA library using NEBNext^®^ Ultra™ RNA Library Prep Kit for Illumina^®^ (NEB, USA). Illumina HiSeq 2500 was used to perform paired-end sequencing of 150 bp reads. The Illumina sequence reads were analyzed for quality control with FastQC (https://www.bioinformatics.babraham.ac.uk/projects/fastqc/), with reads then aligned simultaneously to the human (Ensembl GRCh38.87) and *T. cruzi* (Ensembl Protists ASM20906v1) reference genomes using HISAT2 v2.0.6 (53). The aligned files were ordered and indexed using Samtools v0.1.18 (54) followed by read count calculation using the human genome annotation files of Ensembl genome build GRCh38.87 and the software HTSeq-count v. 0.6.0 (-m intersection-nonempty) (55).

### Transcriptomics Analysis

The relationship among Infected and Control biological replicates of donors A, B, and C for transcriptomes were analyzed through a principal component analysis (PCA) using the ggfortify package R (https://CRAN.R-project.org/package=ggfortify). Differential expression analysis was performed using the DEseq2 package v2.14 from R/Bioconductor (56), comparing infected and control samples (padj ≤ 0.05). All analyses were performed after exclusion of outliers (based on Clustering), leaving at least one representative sample of each donor. Gene ontology enrichment and KEGG Pathway analysis were performed using GOStat (57) and Pathview (58) packages, respectively. Heatmaps for z-score of mean fold change by patient were generated using the gplots package in R (https://CRAN.R-project.org/package=gplots). DEGs were used as input for protein-protein interaction (PPI) analysis using the STRING database (https://string-db.org/) (59). A confidence score of 0.6 was set as a cut-off for protein-protein interaction. The PPI took into account co-expression, co-occurrence, and experimental evidence. The generated interaction networks were uploaded in Cytoscape 3.8.0 for graphical representation (60). DEGs were assigned as functional groups using GO and KEGG databases and specific references.

### Validation

For RT-qPCR, cDNA was synthesized from MoDC RNA and amplified using a GoTaq 2 Step RT qPCR System (Promega) on a StepOnePlus real-time PCR system (Applied Biosystem) and the StepOne™ Software v2.3 (Applied Biosystems), following the manufacturer’s protocol. *B2M* was used as reference gene (61). Specific primers were designed using primer blast (https://www.ncbi.nlm.nih.gov/tools/primer-blast/index.cgi?LINK_LOC=BlastHome). Designed primers: CXCL9-F: 5’-GTGGTGTTCTTTTCCTCTTGGG, CXCL9-R: 5’-CCTTCACATCTGCTGAATCTGG-3’, TNSFS18-F: 5’-GAGATCATCCTGGAAGCTGTGG-3’, TNSFS18-R: 5’-CCAGTCAGACACCTTATTCACG-3’, USP18-F: 5’-ATCCGGAATGCTGTGGATGG-3’, USP18-R: 5’-AGACTCCGTAGATCCAGGAACG-3’. Additionally, RT² qPCR Primers Cat# PPH02447C, PPH02815F, PPH01325A, PPH05983A, and PPH58151A (Qiagen) were used as well. Differential gene expression between Control and Infected group treatments was analyzed by the 2^-ΔΔCT^ method (62).

### Statistical Analysis

All statistical tests were attributed to a p-value ≤ 0.05. Sample raw data distribution was primarily evaluated by the Shapiro-Wilk test to define a statistical test between parametric or non-parametric in each experiment. The Unpaired Student t-test was applied to parametric tests when comparing Infected and Control groups, and for non-parametric tests, the Wilcoxon test was used. GraphPad Prism 6.01 was used for calculations (www.graphpad.com). The statistical details employed in each experiment can be found in the figure legends.

### Data Availability

The accession number for the transcriptome data reported in thispaper is NCBI: GSE158986 (https://www.ncbi.nlm.nih.gov/geo/query/acc.cgi?acc=GSE158986).

## Results

### Metacyclic Trypomastigotes of *T. cruzi* CL Brener Strain Can Infect and Promote Human MoDCs Activation

**Figure 1:**
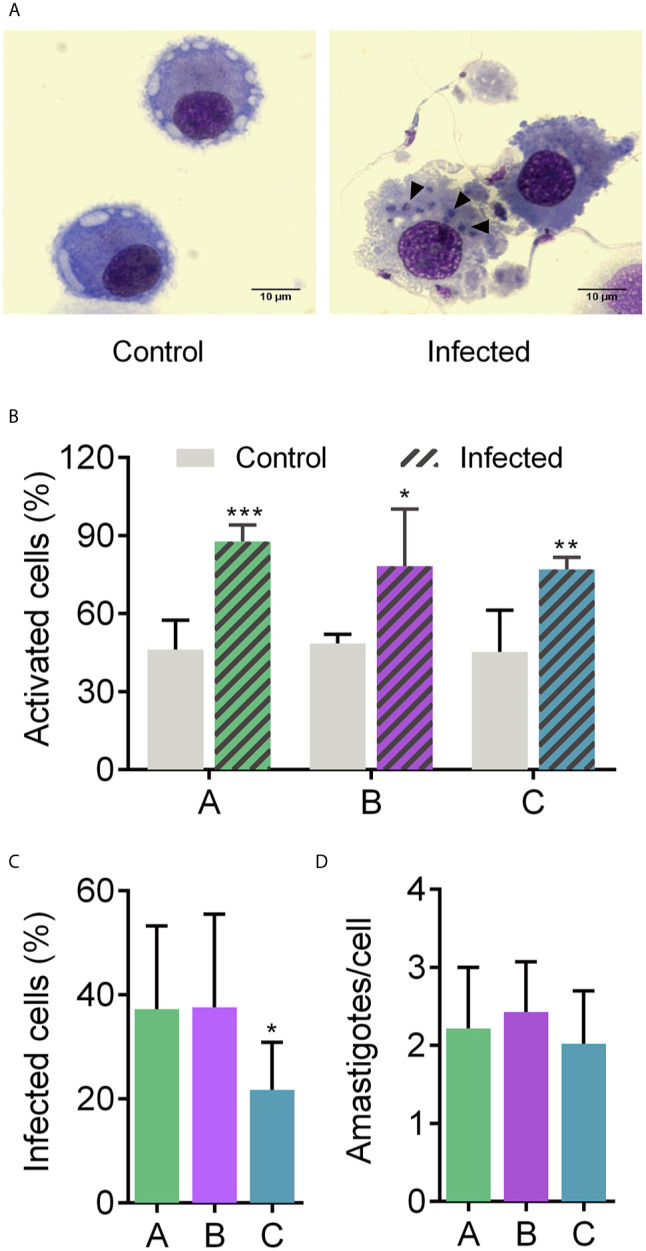
Human MoDCs-*T. cruzi* interaction. Dendritic cells were infected with metacyclic forms of *T. cruzi* for 12 h **(A)** to determine the percentage of activated cells based on the morphology change **(B)**, percentage of infected cells **(C)**, and number of amastigotes per infected cell **(D)**. A, B, and C under X axis refer to donors A, B, and C, respectively. Full arrows: intracellular amastigotes. Mean ± SD, n = 8, double-blind count. Normal distribution was confirmed using Shapiro-Wilk test. Unpaired t-test was used for comparisons. *p < 0.05, **p < 0.01, and ***p < 0.001. Scale bar: 10 µm.

MoDCs from six healthy donors were employed to assess their overall transcriptional response to the presence of *T. cruzi*. Donor characteristics are shown in **Table S1** and **Figure S1**. The morphological changes from peripheral blood monocyte-derived cells, such as cell size increase and the presence of cytoplasmic prolongations, were taken into account to confirm MoDC differentiation (**Figures S2A, B**). Additionally, MoDCs were characterized by flow cytometry, with at least 80% of the population expressing CD1a, CD80, CD86, and CD206 DC markers. Also, CD11c, HLA-DR were detected, in at least 55% of cells (**Figure S2C**). Differentiated MoDCs were placed in contact with *T. cruzi* MTs for 12 h to simulate an early parasite-host cell contact. This time was sufficient for parasite entry (**Figure S3**, **Figure 1A**). The significant morphology change due to the infected condition indicates that interaction with the parasite triggered bystander activation in a heterogeneous population (**Figure 1B**). Interestingly, MoDCs from donor C were significantly less infected than cells from donors A and B, whereas these latter two donors presented a similar infection rate (**Figure 1C**). Despite the difference in the infection rate, the three donors exhibited two amastigotes per infected cell, which may be associated with 12 h incubation and with DC phagocytic features (**Figure 1D**). Thus, the MT forms of CL Brener strain can infect and activate MoDCs, eliciting morphology modifications in the host cell.

### *T. cruzi*-Infection Modulates Gene Expression in MoDCs

**Figure 2:**
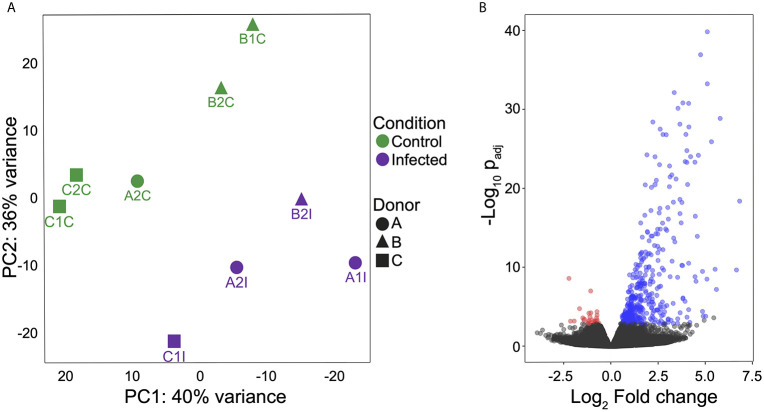
MoDC gene expression profiles during interaction with *T. cruzi* metacyclic forms. **(A)** Principal component analysis (PCA) to evaluate the relationships between control and infected groups. Green: control group and violet: infected group. **(B)** Volcano plot of expression profiles of both groups and DEGs (padj ≤ 0.05) shown according to regulation. Blue: up-regulated, red: down-regulated and black: no significant difference. All analyses were performed after excluding outliers.

RNA-Seq of infected and non-infected MoDCs (used as control) was performed using the Illumina Hi-Seq platform. The experiments were carried out in biological duplicates for each group (n = 3 per group), with an average of 21 million reads obtained for each set. Since each sample from infected cells consisted of a pool of mixed RNAs from MTs and MoDCs, filtered reads were simultaneously mapped onto both human and *T. cruzi* genomes to avoid incorrect mapping of parasite genes onto the human genome (**Table S2**). From infected samples, 2 to 21% of total reads mapped onto the parasite genome. On the other hand, non-infected MoDCs presented less than 0.02% of the total mapped reads miss-mapping onto the *T. cruzi* genome (**Table S3**). A total of 16,264 human genes were annotated at least once in the samples (GEO accession GSE158986), representing more than 76% of the total known protein-coding genes in the human genome (63), and indicating high breadth of coverage for the transcriptome. A clear separation between infected and control samples was obtained in a principal component analysis (PCA) after the removal of three outliers (**Figure 2A**). A total of 468 DEGs were identified, with 439 up-regulated and 29 down-regulated (**Figure 2B** and **Table S4**). These results show that *T. cruzi* infection modulates the gene expression profile of MoDCs during a simulated first contact and that a modulation signature can be observed, despite the natural variation among donors.

### Response to Virus as the Most Represented Biological Function

**Figure 3:**
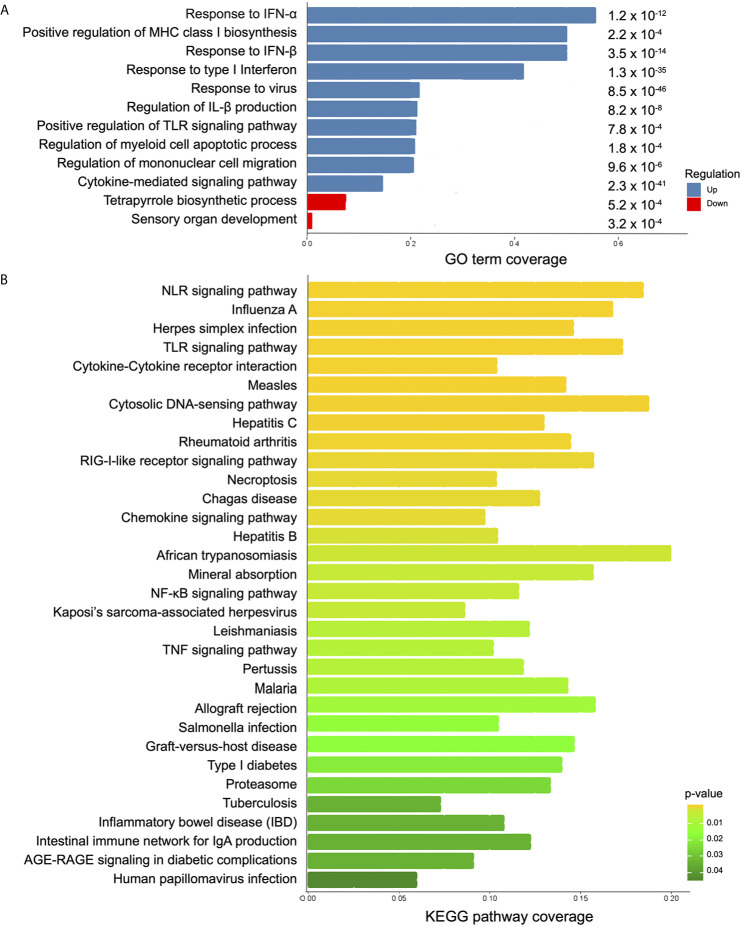
Analysis of biological function in differentially expressed genes between infected and control MoDCs. **(A)** A summary of the enriched biological processes from GO is shown as coverage (number of DEGs per total number of genes in each GO term). Blue bars: up-represented processes and red bars: down-represented processes. p-value is displayed at the end of each bar. **(B)** KEGG enriched pathways are represented as KEEG coverage (DEGs per total number of genes in each KEGG pathway). Bar colors indicate p-values.

To identify known cellular processes and functional annotations associated with the transcriptome presented here, the resulting DEGs were analyzed using Gene Ontology (GO) and Kyoto Encyclopedia of Genes and Genomes (KEGG) databases. GO enrichment analysis revealed 517 up- and 2 down-represented terms (**Table S5**). In a more comprehensive analysis, taking into account p-value ≤ 0.0008 coverage and non-redundant GO terms, the top 10 up-represented terms were selected (**Figure 3A**). Response to type I interferon, response to virus, MHC class I processing, and some PRRs signaling were the most important terms among the up-regulated genes. Expression levels of genes encoding proteins known to be important for inflammasome activation (e.g., RIG-I, IFI16, and AIM2) in response to virus were significantly enriched (p-value 0.00009). Regarding KEGG enrichment analysis, 32 differentially regulated pathways were seen among the samples (**Table S6**). Among the highlighted KEGG terms, multiple PRR signaling pathways were found, including inflammasomes (NLRs and cytosolic DNA sensors), and disease-specific pathways such as measles, herpes, hepatitis, leishmaniasis, and African trypanosomiasis (**Figure 3B**). Interestingly, viruses were included in 28% of the KEGG pathways enriched terms. Taken together the enrichment analyses, it seems that antiviral response may have an important function in the early *T. cruzi* infection.

**Figure 4:**
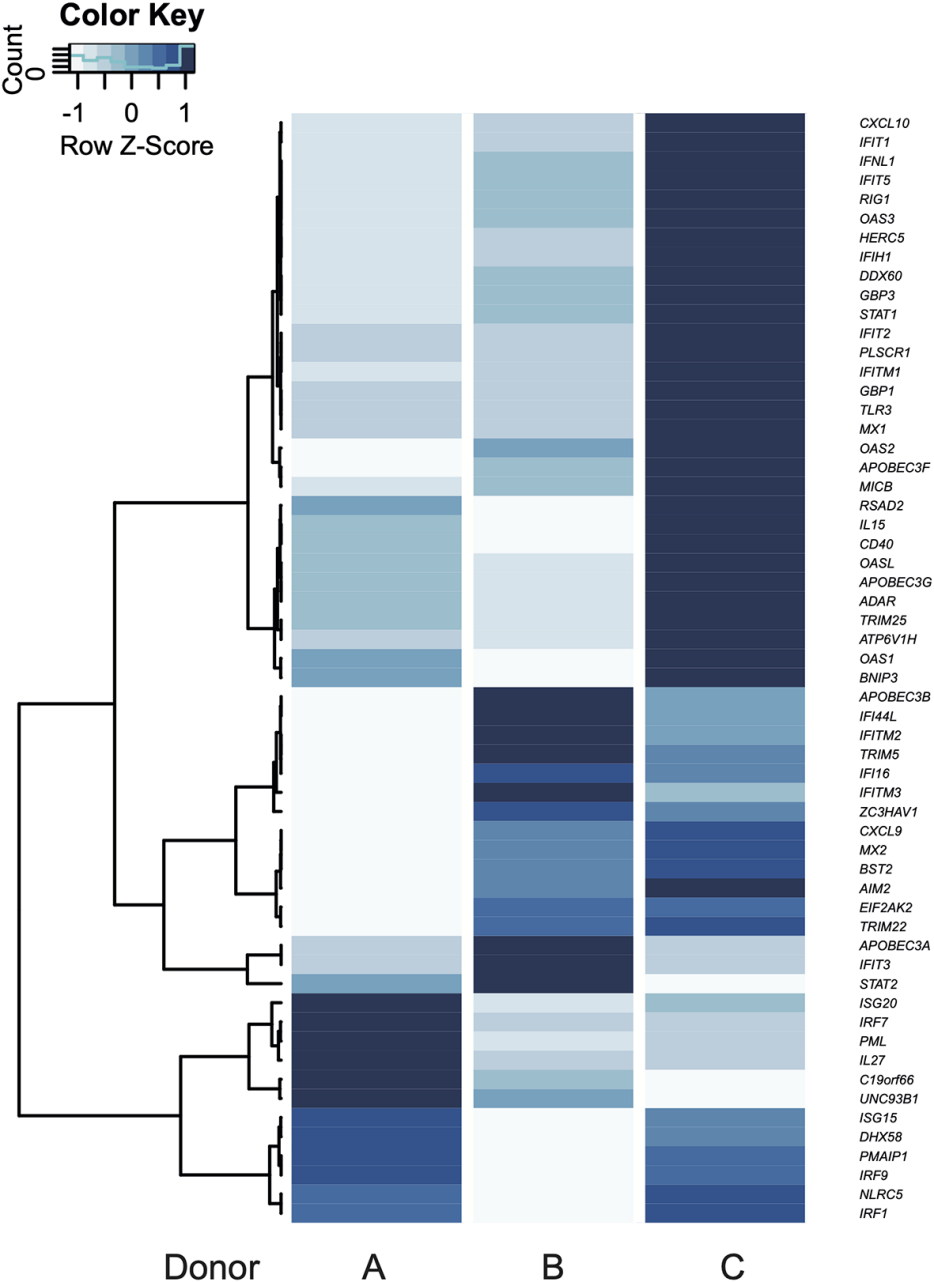
Individual gene expression patterns according to GO terms. Response to virus is shown in a heatmap. Blue color scale: variation of mean fold change from each gene (Z-Score). Every gene in the heatmaps was up-regulated in the transcriptome.

To compare the intra-donor expression pattern from the up-regulated genes belonging to “defense response to virus” (GO:0051607) term, a heat map was elaborated (**Figure 4**). Donor C showed an above-average expression in 78% of the genes, with the exception of *APOBEC3B*, *IFIT3*, *UNC93B1*, *C19orf66*, *IL27*, *PML*, *IRF7*, *ISG20*, and *STAT2*. On the other hand, the latter seven genes were expressed above average in donor A, which has an above-average expression in 21% of the genes. Donor B presented an above-average expression in 31% of the genes (**Figure 4**, GEO accession GSE158986). Notably, the genes coding for the inflammasome sensor proteins AIM2 and IFI16 were expressed above the average in donors B and C, while *RIG-I* was expressed above average only in C.

### Virus Response as a Central Pathway in the Protein-Protein Interaction Network

**Figure 5:**
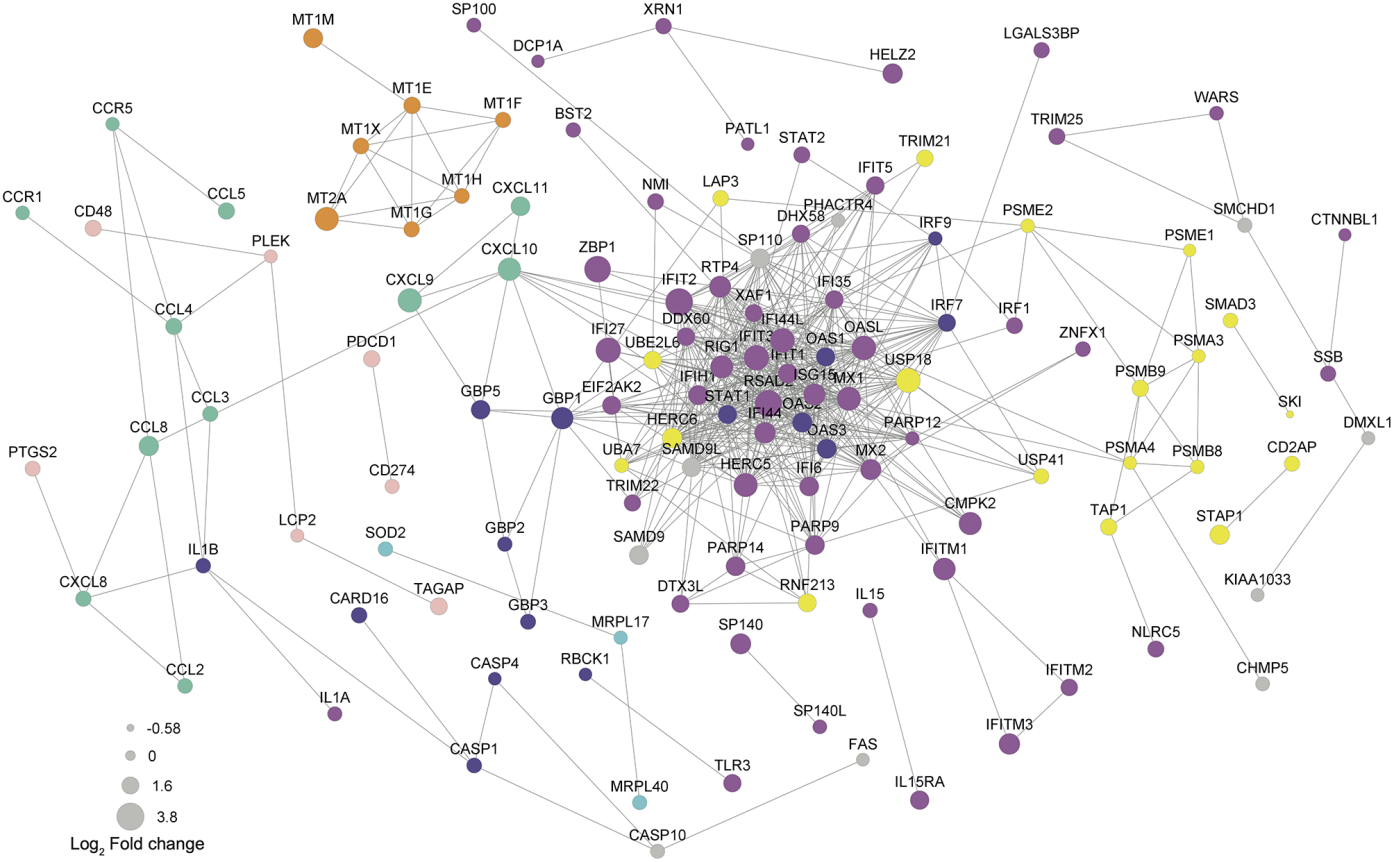
Protein-protein interaction underlines genes from virus response as network-hubs. DEGs were used as input for protein-protein interaction (PPI) analysis in STRING database. Magenta 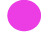: virus response (GO:0009615); yellow 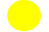: exogenous antigen processing and presentation (GO:0002479) including proteasome (hsa03050) and ubiquitination/deubiquitination (GO:0016567/GO:0016579); violet 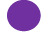: NOD-like receptor signaling pathway (hsa04621); mint green 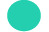: chemokine signaling pathway (hsa04602); shell pink 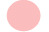: lymphocyte co-stimulation; orange 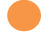: metallothioneins related to virus response; cyan 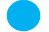: protein related to reactive oxygen species. A confidence score of 0.6 was set as a cut-off allowing co-expression, co-occurrence, and experimental as evidences. Edge lengths represent the STRING score of evidence: higher scores reflect shorter lengths.

In order to determine biological significance in controlling the magnitude of differential expression of each gene, a protein-protein interaction (PPI) network of DEGs was generated using the STRING database. After excluding unannotated genes and proteins with no reported interactions, the 126 proteins participating in the network presented seven consistent functional patterns (**Figure 5**). Remarkably, four of them are closely related to virus response: Virus response (magenta), MHC-I antigen processing and presentation through ubiquitin-proteasome (yellow), NOD-like receptor signaling pathway (violet), and metallothioneins (orange). Moreover, three typical DC functions were up-regulated and highlighted in the PPI network: reactive oxygen species production (cyan), lymphocyte recruitment (mint green), and co-stimulation (shell pink). Regarding the virus-related response, a direct correlation between fold change and PPI probability (represented by diameter and edge length, respectively) was observed. Noteworthy, the top 10 network-hubs: ISG15, IFIT3, MX1, RSAD2, STAT1, IFIH1, OASL, IFIT1, IFI44L, and IFI44 belong to the “response to virus” GO term, reinforcing that *T. cruzi* may activate a virus-related response in MoDCs. This pattern indicates that the differential expression obtained through the transcriptome analysis is not random and supports a biological significance for the triggered pathways. Within the PPI network, the virus response pathway was connected to NOD-like receptor signaling, lymphocyte recruitment, and MHC-I antigen processing and presentation pathways through GBP1, CXCL10, and USP18, respectively. Furthermore, CCL4 links the lymphocyte recruitment cluster to the co-stimulation-related proteins. Finally, metallothioneins clustered into a single group that presents a strong PPI probability.

### Transcriptome Validation

**Figure 6:**
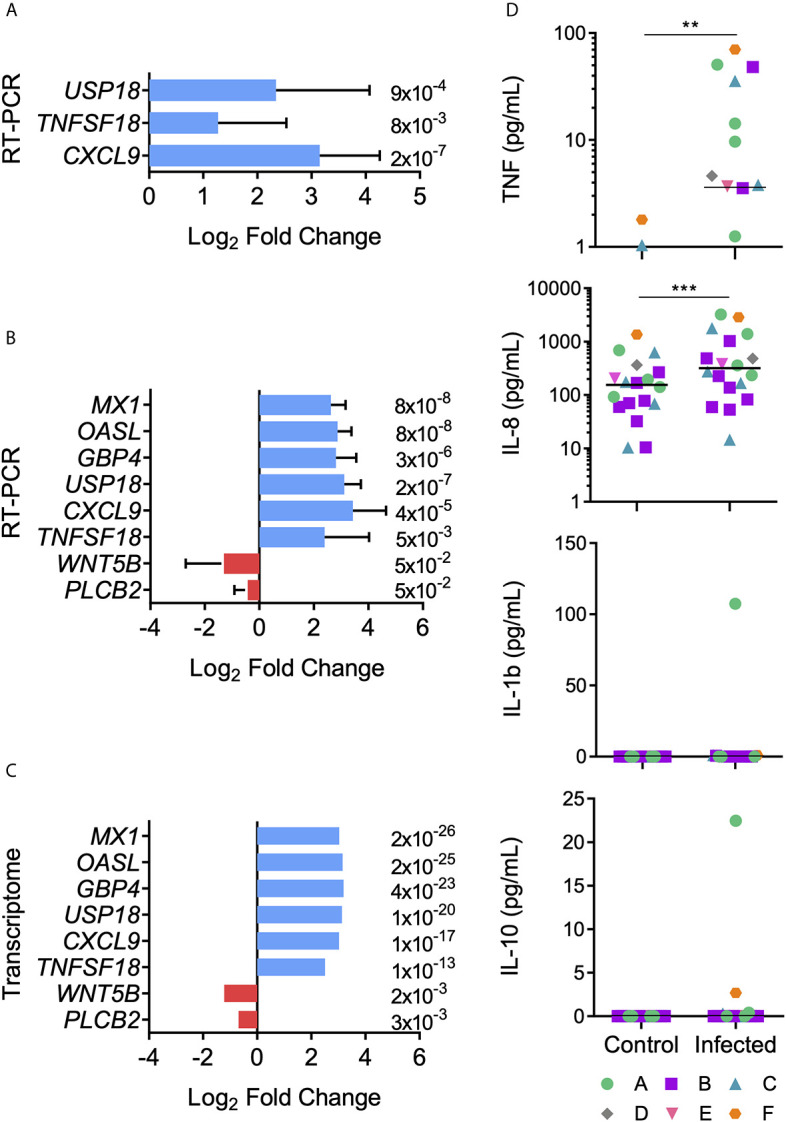
Transcript and protein expression levels after MoDCs and *T. cruzi* first contact. Differential expression of genes was confirmed by RT-qPCR. **(A)** Mean Log_2_ Fold change ± SD was calculated using *USP18*, *TFSF18*, and *CXCL9* genes from donors A, B, and C in triplicate (n = 9). **(B)** Mean Log_2_ Fold change ± SD was calculated using *USP18*, *TFSF18*, *CXCL9*, *WTN5B*, *PLCB2*, *OASL*, *MX1*, and *GBP4* genes from the six donors (n = 6). **(C)** Transcriptome Log_2_ Fold change is shown for comparisons. Red: down-regulated genes, blue: up-regulated genes. Data from panels A and B present a normal distribution according to Shapiro-Wilk test. Unpaired t-test was used for comparisons. p-value is displayed at the end of each bar. **(D)** Control and infected MoDC culture supernatants were collected after 12 h-interaction assays. TNF, IL-8, IL-1β, and IL-10 cytokine concentrations were measured using a cytometric bead array kit. Horizontal lines inside the graphics represent median values and each point represents a replicate (n = 19). Data from panel D did not present a normal distribution according to Shapiro-Wilk test. Significant differences were tested using the Mann Whitney test. **p < 0.01; ***p < 0.001.

RT-qPCR validation assays were performed for three up-regulated genes chosen from the RNA-seq data as being related to the DC function of antigen presentation: *USP18*, *TNFSF18*, *CXCL9*. Three independent infections from each donor (A, B and C) were carried out and expression modulation evaluated in the selected genes. The analysis of these nine samples presented concordance between RNA-seq and RT-qPCR data, thus validating the RNA-seq results (**Figure 6A**). Our next goal was to determine if other donors would present the same expression signature found in the transcriptomic data demonstrated here. Therefore, MoDCs from three new donors (D, E, and F) were obtained, infected with *T. cruzi*, and analyzed by RT-qPCR. To reinforce our validation assay, five modulated genes were included in this analysis: *MX1*, *OASL*, *GBP4*, *WNT5B*, *PLCB2* (**Figures 6B,C**). These genes were selected from Human papillomavirus infection (hsa:05165), Influenza A (hsa:05164), and NOD-like receptor signaling (hsa:04621) KEGG pathways, including up- and down-regulated genes. Compatible with the previous results, with fold changes and p-values closely related to the transcriptome data, we observed concordance between both techniques. Together, these two analyses validate the obtained transcriptome among our original three donors, with the same patterns presented.

### Proinflammatory Cytokines Characterize the Early Contact Between DCs and *T. cruzi*

According to the presented transcriptome, cytokine and chemokine genes, such as *TNF*, *IL-1β*, *IL-1α*, *IL-15*, and *IL-8*, were up-regulated after the MoDCs-*T. cruzi* interaction. Among the anti-inflammatory cytokines, only *IL-10* was up-regulated, with a fold change close to 2 and a q-value of 0.009 (see **Table S4**). These results provide evidence for a tendency of proinflammatory cytokine production after the first 12 h of interaction between DCs and *T. cruzi*. To investigate if the transcriptome-predicted proinflammatory milieu is reflected at a protein level, innate immune cytokines were measured in the culture supernatants, with a significant increase in the production of TNF and IL-8 observed in the infected samples (**Figure 6D**). On the other hand, no significant difference was detected in the production of IL-1β and IL-10 (**Figure 6D**).

## Discussion

Early immunological events on Chagas disease are the result of diverse molecular pathways modulated by a combination of *T. cruzi* and host factors, such as secreted and cell surface polymorphic molecules (64). In this work, we revealed a set of genes modulated in human dendritic cells after contact with *T. cruzi* metacyclic forms, since both cells represent the first actors in a natural infection that can affect the progress of the disease. It is worth to note that MTs obtained under chemically conditions are capable of reproducing the parasite biological behavior (47). Since control MoDCs presented a relatively high expression of typical activation markers, morphological change is a useful tool for assessing maturation of DCs (36, 51, 52). Using this approach, a high percentage of morphological changes was observed in the MoDCs, indicating bystander activation after contact with the parasite (65–68), and a similar infection rate to those seen in other studies (39, 40). A mixed gene expression profile from bystander and infection activation in MoDCs is the expected scenario in the initial stage of natural infection when not every cell is infected or mature (35).

**Figure 7:**
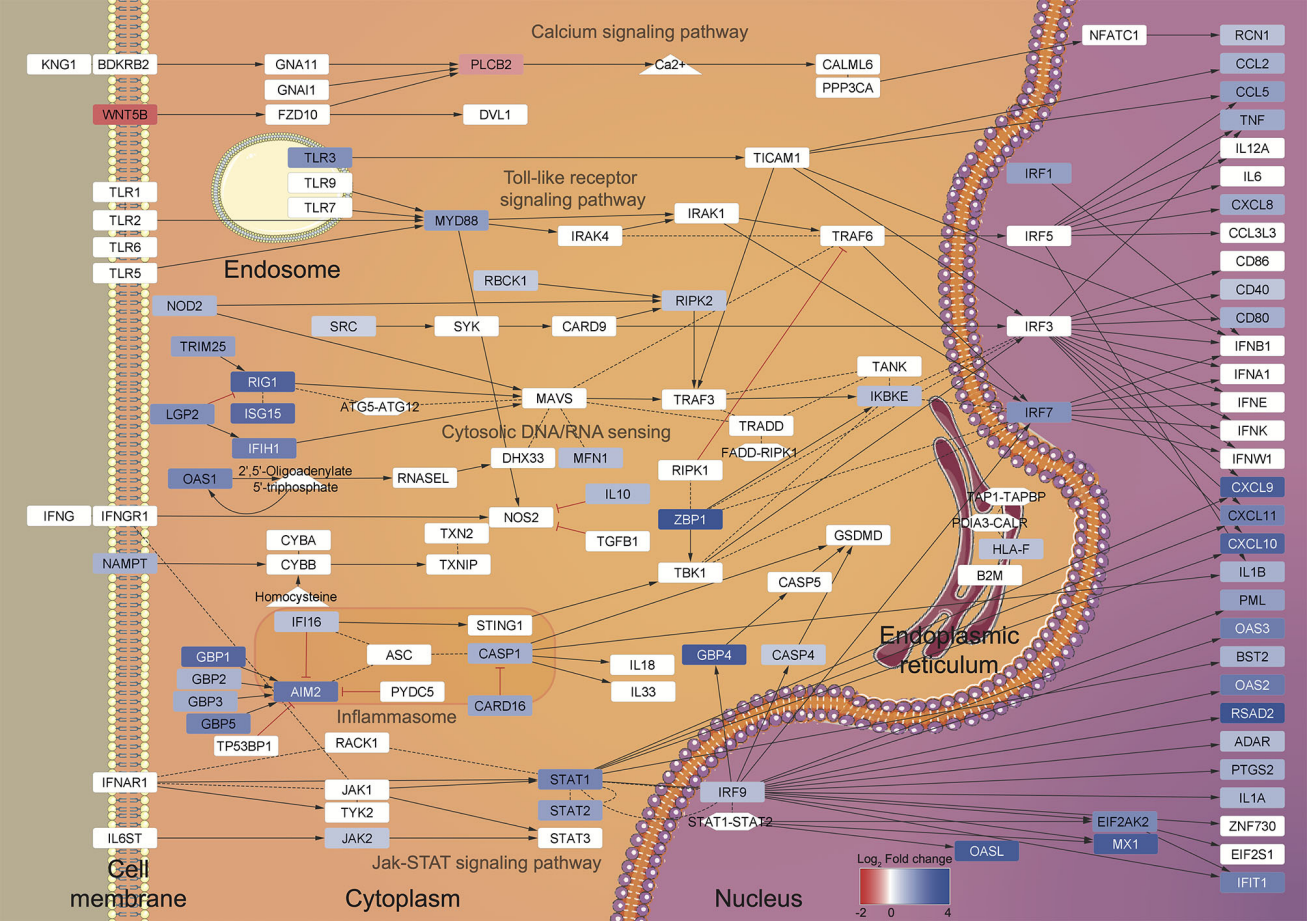
Summary of the regulated pathways during DCs-*T. cruzi* first contact. A view of the gene expression patterns was generated using the transcriptome data and the enriched KEGG pathways as input for BioNSi in Cytoscape v.3.8.0. The KEGG pathways (named in gray) are displayed according to the transcriptome Log_2_ Fold change scale (blue: up-regulated genes, red: down-regulated genes) and cellular localization.

Using this RNA-seq approach, we identified 468 DEGs from three healthy female donors that shared a gene expression pattern. Our main finding was that, during the initial contact with *T. cruzi*, MoDCs trigger a response similar to that of a viral infection, including expression of several type I interferon-induced genes derived from JAK/STAT signaling pathway (69, 70) and viral inflammasome activation (**Figure 7**). Transcriptome analyses using human foreskin fibroblasts infected by Y strain (71) or Sylvio strain (72) blood trypomastigotes showed induction of cytokine production and enhanced expression of type I interferon-inducible genes. These two studies corroborate our results regarding the activation of a viral response in human cells infected by *T. cruzi*, although they did not explore those findings. The immune aspect was explored using human monocyte-derived macrophages infected by two *Leishmania* species (73). In this case, the results were filtered for phagocytosis-related genes, and among the up-regulated genes, inflammatory IL-1β, IL-6, and TNF cytokines, and metallothionein 1 family members were highlighted. Additionally, 16 of the 39 KEGG enriched pathways observed after *Leishmania* infection are identical to those obtained in our study, including herpes simplex, hepatitis C and B, influenza A virus, and measles pathways (73).

Currently, the immune response to viruses is well elucidated, and it is known that besides limiting viral replication (74), type I IFN regulates the activation of NK cytotoxic function (75), synergizes with TNF to induce iNOS in macrophages (76), and induces DC maturation (77). Notwithstanding, there are no studies that associate human Chagas disease acute phase and type I interferon-inducible genes or that directly correlate the host response during the parasite infection with a virus-like response. Only a few studies using the murine model have shown the importance of type I IFNs during parasite infection. For instance, mouse NK cells lacking IFN-α/β receptor were unable to activate their cytotoxic function (78), and murine macrophages seem to need type I IFN co-stimulation for nitric oxide production (79). Also, MyD88/TRIF deficient mice did not produce IFN-β and could not control *T. cruzi* proliferation (80). Interestingly, type I IFN released at the parasite inoculation site seems to be strain-dependent, since less infective strains were incapable of triggering the cytokine production (81).

Similar to antiviral pathways, inflammasome activation has been widely studied in mammal models, revealing a fundamental role in signal transduction and pyroptosis, a proinflammatory form of cell death (82). Among the inflammasome sensor proteins, NOD1, NOD2, and NLRP3 have been studied in the host-*T. cruzi* context, with apparent contradictory results regarding their effective roles in the protection against infection (83–86). Interestingly, we have identified up-regulation of the virus-related IFI16, RIG-I, and AIM2 sensor proteins, which are known to drive the maturation and secretion of IL-1β after foreign RNA/DNA recognition (87–89). In *Plasmodium berghei* and *Aspergillus fumigatus*, AIM2 and NLRP3 may function in a redundant manner to trigger inflammasome activation and, consequently, aid in pathogen infection control (90, 91). Additionally, in *Leishmania* and *Toxoplasma*, inflammasome activation can lead to parasite clearance, host susceptibility or even an exacerbated immune response (92). Nevertheless, the role of inflammasomes and their relationship to Chagas disease need to be further explored.

According to our PPI analysis, the genes belonging to virus response show robust evidence for interaction, operating as hubs in the network (**Figure 5**, magenta), and indicating the importance of this pathway during the early *T. cruzi* infection. Moreover, other enriched pathways in the PPI network are also closely related to virus response. For example, viral antigens are usually presented to T lymphocytes by MHC-I molecules after ubiquitin-proteasome degradation (**Figure 5**, yellow) (93). Equally, metallothioneins (**Figure 5**, orange) are reported to be modulated during viral infection or cytokine stimulus, regulating host metal ion release, redox status, enzymatic function, and cell signaling (94). Additionally, reactive oxygen species production (**Figure 5**, cyan) and T lymphocyte recruitment and co-stimulation (**Figure 5**, mint green and shell pink, respectively) are known to be essential processes in both viral and *T. cruzi* infection (95–98).

Inflammatory cytokines such as IL-8, TNF, IL-12, and IL-1β, are the typical cocktail (99) that contribute to *T. cruzi* elimination (10, 43, 84, 100, 101). Looking at the secreted set of cytokines after 12 h of MoDCs-*T. cruzi* metacyclic interaction, an increment of IL-8 and TNF were detected as early products of gene transcription, while IL-12 expression did not change at transcriptional or protein level. MoDCs presented a reduced production of IL-12 and TNF-α after 24 h of infection with the Tehuantepec strain (39), while BM-DCs showed diminished IL-12 secretion but higher IL-10 production 24 h post-infection (102). On the other hand, IL-10 and IL-1β transcripts were up-regulated, but these proteins were not detected in culture supernatants. In the case of IL-1β, CARD16, a protein reported as an inhibitor of the caspase-1 activation, was found up-regulated at the transcriptional level (103, 104). Without caspase-1 activity, pro-IL-1β is not cleaved into an active IL-1β, which could be one of the reasons for its absence in culture supernatants. Concerning IL-10, it is classically produced upon activation of type I-IFN response (105), and also induced through NOD2, here up-regulated, along with TLRs (106–110). However, IL-10 transcript contains multiple copies of destabilizing motifs and its stability is affected by several factors, including IL-10 itself (111–113), which could explain a rapid IL-10 transcription but a short-lasted or even null secretion.

Despite the viral-related response being common among the donors, donor C showed the lowest infection rates, and this is probably related to the set of genes that were expressed above or below average in this donor. Nevertheless, the high variations among experiments in the human model make the evaluation of these differences difficult at the transcript or protein level. Within the “defense response to virus” GO term, donor C showed an above-average expression in 45 of the 58 total regulated genes. This stronger expression in the virus response genes could be the explanation for the lower infection in donor C. Given the supposed importance of a viral immune response during the host’s first contact with the parasite, one could hypothesize that donor C’s longer history of viral immunizations may confer some form of protection against *T. cruzi* infection. This type of nonspecific protection, provided by commercial vaccines, has recently been explored and is known as trained innate immunity, where epigenetic changes in innate immune cells can be observed after vaccination and may prepare them to respond to a different pathogen (114, 115).

To validate the transcriptome and increase the studied individuals, some relevant regulated genes were selected. CXCL9 (fold change = 8.1) is a chemokine mainly produced by monocytes, epithelial cells, fibroblasts, and tumor cells, which attracts T lymphocytes to the infection site (116). TNFSF18 (fold change = 5.7) is a molecule mainly expressed by antigen-presenting cells that enhances DC and lymphocyte activation (117). USP18 (fold change = 8.7) is a cysteine protease that negatively regulates type I IFN signaling, promoting CD11b^+^ DC differentiation (118). Then, these three proteins seem to be important during early infection, when DCs need to differentiate to present antigens and recruit T cells, and they were consistently more expressed in the infected MoDCs from six donors. Additionally, the regulation of *GBP4*, *MX1*, *OASL*, *PLCB2*, and *WNT5B*, genes related to the virus response, was consistent among the donors. GBP4 (fold change = 9.2) is a IFN-γ inducible GTPase containing a C-terminal domain that enable inflammasome activation to eliminate intracellular bacteria and virus (119–121). MX1 (fold change = 8) is also a GTPase that blocks viral replication, and is widely studied in the Influenza infection (122, 123). OASL (fold change = 8.9) is a type I IFN inducible protein that inhibits viral replication during the early phase of infection by degrading viral RNA (124, 125). Therefore, our data show that virus-related inflammasome induction is important during the first contact between DCs and *T. cruzi*, and this response is more similar to that directed to RNA viruses. On the other hand, *PLCB2* (fold change = − 0.6) and *WNT5B* (fold change = −0.4) were down-regulated in transcriptional levels. WNT5B is correlated with hematopoietic/bone marrow cell differentiation GM-CSF-induced and to proinflammatory cytokine regulation during infections (126, 127). PLCB2 negatively regulates the proinflammatory response produced by viral infections (128). Down-regulated expression of *WNT5B* and *PLCB2* was expected. Further studies are required to test if other strains of *T. cruzi* up-regulate the same gene set, or if a strain of different infection capacity will induce different gene expression patterns that can be associated with pathogeny.

A vast range of pathogens, from viruses to multicellular parasites, may also shape DC metabolism and immune functions leading towards a tolerogenic phenotype. Hepatitis C virus (HCV) NS3 and HCV core proteins can inhibit DC differentiation *via* an IL-10 induction (129). Moreover, DCs recovered from patients infected with hepatitis B virus exhibited decreased antigen-presenting, migration, and cytokine production capacity (130). Human DC exposure to *Mycobacterium tuberculosis* resulted in the downregulation of CD11a, CD11b, and CD18 expression with compromised DC adherence to endothelial cells and migration toward a chemokine gradient (131). Finally, *Plasmodium falciparum* binds to the surface of myeloid DCs and deeply reduces the expression of MHC class II, CD83 and CD86 co-stimulatory molecules, and maturation of DCs (132). However, the delimitated conditions (*in vitro*, 12 h after infection, parasite strain, DC subset) in which we carried out our tests do not allow us to conclude whether the antiviral response will be exclusively to the detriment or the benefit of the parasite.

In conclusion, our transcriptome has brought to light virus response as an important and unexplored process during the first hours of *T. cruzi*-host interaction. These discoveries open up new perspectives for the study of this disease, with better-elucidated viral infection mechanisms expanding current knowledge. Future work regarding the role of the type I interferon-induced genes for antigen presentation and T lymphocyte activation should be conducted to elucidate the immune response against *T. cruzi* and disease progression.

## Data Availability Statement

The datasets generated for this study can be found in NCBI: GSE158986 (https://www.ncbi.nlm.nih.gov/geo/query/acc.cgi?acc=GSE158986).

## Ethics Statement

The studies involving human participants were reviewed and approved by the Research Ethics Committee from the Medicine Faculty (Comitê de Ética em Pesquisa da Faculdade de Medicina)–The University of Brasília (CAAE: 54822616.7.0000.5558). The patients/participants provided their written informed consent to participate in this study.

## Author Contributions

Conceptualization, NG-J, AR, FM, IB, and JS. Methodology, NG-J, AR, TR, FM, CF, MB, IB, and JS. Software, TR and NG-J. Formal analysis, NG-J, AR, and TR. Investigation, NG-J and AR. Resources, CF, IB, and JS. Data curation, NG-J, AR, and TR. Writing—original draft, NG-J, AR, and FM. Writing—review and editing, NG-J, FM, TR, CF, MB, IB, and JS. Visualization, NG-J and IB. Supervision, CF, MB, IB, and JS. Funding acquisition, IB and JS. All authors contributed to the article and approved the submitted version.

## Funding

This work was supported by grants and fellowships awarded by the Fundação de Amparo à Pesquisa do Distrito Federal (FAP-DF, 0193.001803/2017 and 0193.001485/2017), Coordenação de Aperfeiçoamento de Pessoal de Nível Superior (CAPES, grant 923/18 CAPES-COFECUB), Conselho Nacional de Desenvolvimento Científico e Tecnológico (CNPq, INCT-MCTI/CNPq/FAPs 16/2014). NG-J and AR received scholarships from CNPq and CAPES. The funders had no role in study design, data collection and interpretation, or the decision to submit the work for publication.

## Conflict of Interest

The authors declare that the research was conducted in the absence of any commercial or financial relationships that could be construed as a potential conflict of interest.

## References

[1] World Health Organization [WHO]. Chagas disease (American trypanosomiasis). World Health Organization [WHO] (2020). Available at: https://www.who.int/health-topics/chagas-disease. PMC2305660 10063697

[2] Pan American Health Organization [PAHO]. Chagas in the Americas for Public Health Workers. Pan American Health Organization [PAHO] (2017). Available at: https://www.paho.org/en/documents/chagas-americas-public-health-workers-2017.

[3] World Health Organization [WHO]. Global distribution of cases of Chagas disease, based on official estimates, 2018. World Health Organization [WHO] (2018). Available at: https://www.who.int/docs/default-source/ntds/chagas-disease/chagas-2018-cases.pdf?sfvrsn=f4e94b3b_2.

[4] Schmunis GA Yadon ZE. Chagas disease: A Latin American health problem becoming a world health problem. Acta Trop (2010) 115:14–21. 10.1016/j.actatropica.2009.11.003 19932071

[5] Imai K Maeda T Sayama Y Osa M Mikita K Kurane I. Chronic Chagas disease with advanced cardiac complications in Japan: Case report and literature review. Parasitol Int (2015) 64:240–2. 10.1016/j.parint.2015.02.005 25744336

[6] Rios LE Vázquez-Chagoyán JC Pacheco AO Zago MP Garg NJ. Immunity and vaccine development efforts against Trypanosoma cruzi. Acta Trop (2019) 200:105168. 10.1016/j.actatropica.2019.105168 31513763 PMC7409534

[7] Rassi AJ Rassi A Marcondes De Rezende J. American Trypanosomiasis (Chagas Disease). Infect Dis Clin North Am (2012) 26:275–91. 10.1016/j.idc.2012.03.002 22632639

[8] Rassi A Rassi A Marin-Neto JA. Chagas disease. Lancet (2010) 375:1388–402. 10.1016/S0140-6736(10)60061-X 20399979

[9] Bern C. Chagas’ Disease. N Engl J Med (2015) 373:456–66. 10.1056/NEJMra1410150 26222561

[10] Andrade DV Gollob KJ Dutra WO. Acute Chagas Disease: New Global Challenges for an Old Neglected Disease. PLoS Negl Trop Dis (2014) 8:e3010. 10.1371/journal.pntd.0003010 25077613 PMC4117453

[11] Bonney KM Engman DM. Autoimmune pathogenesis of chagas heart disease: Looking back, looking ahead. Am J Pathol (2015) 185:1537–47. 10.1016/j.ajpath.2014.12.023 PMC4450315 25857229

[12] Chagas C. Nova tripanozomiaze humana: estudos sobre a morfolojia e o ciclo evolutivo do Schizotrypanum cruzi n. gen., n. sp., ajente etiolojico de nova entidade morbida do homem. Mem Inst Oswaldo Cruz (1909) 1:159–218. 10.1590/S0074-02761909000200008

[13] Shikanai-Yasuda MA Carvalho NB. Oral transmission of Chagas disease. Clin Infect Dis (2012) 54:845–52. 10.1093/cid/cir956 22238161

[14] Yoshida N. Molecular mechanisms of Trypanosoma cruzi infection by oral route. Mem Inst Oswaldo Cruz (2009) 104:101–7. 10.1590/S0074-02762009000900015 19753464

[15] de Lana M Marques E Machado DM. Biology of Trypanosoma cruzi and Biological Diversity. 1st ed. Amsterdam: Elsevier Inc (2010). 10.1016/B978-0-12-384876-5.00014-9

[16] Pérez-Molina JA Molina I. Chagas disease. Lancet (2018) 391:82–94. 10.1016/S0140-6736(17)31612-4 28673423

[17] Monteon V. Trypanosoma cruzi: the early contact between insect-derived metacyclic trypomastigotes and the mammalian cells. Ann Parasitol (2019) 65:193–204. 10.17420/ap6503.201 31578843

[18] Giordano R Fouts DL Tewari D Colli W Manning JE Alves MJM. Cloning of a surface membrane glycoprotein specific for the infective form of Trypanosoma cruzi having adhesive properties to laminin. J Biol Chem (1999) 274:3461–8. 10.1074/jbc.274.6.3461 9920891

[19] Scharfstein J Schmitz V Morandi V Capella MMA Lima APCA Morrot A. Host cell invasion by Trypanosoma cruzi is potentiated by activation of bradykinin B2 receptors. J Exp Med (2000) 192:1289–99. 10.1084/jem.192.9.1289 PMC2193362 11067878

[20] Turner CW Lima MF Villalta F. Trypanosoma cruzi uses a 45-kDA mucin for adhesion to mammalian cells. Biochem Biophys Res Commun (2002) 290:29–34. 10.1006/bbrc.2001.6189 11779128

[21] Herrera EM Ming M Ortega-Barria E Pereira ME. Mediation of Trypanosoma cruzi invasion by heparan sulfate receptors on host cells and penetrin counter-receptors on the trypanosomes. Mol Biochem Parasitol (1994) 65:73–83. 10.1016/0166-6851(94)90116-3 7935630

[22] Alves M Colli W. “Role of the gp85/Trans-Sialidase Superfamily of Glycoproteins in the Interaction of Trypanosoma cruzi with Host Structures”. In: Molecular Mechanisms of Parasite Invasion. New York: Springer (2008). p. 58–69 . 10.1007/978-0-387-78267-6_4 18512341

[23] Bafica A Santiago HC Goldszmid R Ropert C Gazzinelli RT Sher A. Cutting Edge: TLR9 and TLR2 Signaling Together Account for MyD88-Dependent Control of Parasitemia in Trypanosoma cruzi Infection. J Immunol (2006) 177:3515–9. 10.4049/jimmunol.177.6.3515 16951309

[24] Campos MA Almeida IC Takeuchi O Akira S Valente EP Procopio DO. Activation of Toll-like receptor-2 by glycosylphosphatidylinositol anchors from a protozoan parasite. J Immunol (2001) 167:416–23. 10.4049/jimmunol.167.1.416 11418678

[25] Calderón J Maganto-Garcia E Punzón C Carrión J Terhorst C Fresno M. The receptor Slamf1 on the surface of myeloid lineage cells controls susceptibility to infection by Trypanosoma cruzi. PLoS Pathog (2012) 8:e1002799. 10.1371/journal.ppat.1002799 22807679 PMC3395606

[26] Medeiros MM Peixoto JR Oliveira A-C Cardilo-Reis L Koatz VLG Van Kaer L. Toll-like receptor 4 (TLR4)-dependent proinflammatory and immunomodulatory properties of the glycoinositolphospholipid (GIPL) from Trypanosoma cruzi. J Leukoc Biol (2007) 82:488–96. 10.1189/jlb.0706478 17540734

[27] Schmitz V Almeida LN Svensjö E Monteiro AC Köhl J Scharfstein J. C5a and bradykinin receptor cross-talk regulates innate and adaptive immunity in Trypanosoma cruzi infection. J Immunol (2014) 193:3613–23. 10.4049/jimmunol.1302417 25187655

[28] Poncini CV Giménez G Pontillo CA Alba-Soto CD de Isola ELD Piazzón I. Central role of extracellular signal-regulated kinase and Toll-like receptor 4 in IL-10 production in regulatory dendritic cells induced by Trypanosoma cruzi. Mol Immunol (2010) 47:1981–8. 10.1016/j.molimm.2010.04.016 20537708

[29] Araujo FF Gomes JAS Rocha MOC Williams-Blangero S Pinheiro VM Morato MJF. Potential role of CD4 +CD25 HIGH regulatory T cells in morbidity in Chagas disease. Front Biosci (2007) 12:2797–806. 10.2741/2273 17485260

[30] Souza PEA Rocha MOC Rocha-Vieira E Menezes CAS Chaves ACL Gollob KJ. Monocytes from patients with indeterminate and cardiac forms of Chagas’ disease display distinct phenotypic and functional characteristics associated with morbidity. Infect Immun (2004) 72:5283–91. 10.1128/IAI.72.9.5283-5291.2004 PMC517423 15322024

[31] de Araújo FF Corrêa-Oliveira R Rocha MOC Chaves AT Fiuza JA Fares RCG. Foxp3 +CD25 high CD4 + regulatory T cells from indeterminate patients with Chagas disease can suppress the effector cells and cytokines and reveal altered correlations with disease severity. Immunobiology (2012) 217:768–77. 10.1016/j.imbio.2012.04.008 22672991

[32] Dutra WO Menezes CAS Magalhães LMD Gollob KJ. Immunoregulatory networks in human Chagas disease. Parasite Immunol (2015) 36:377–87. 10.1016/j.micinf.2011.07.011.Innate PMC4143493 24611805

[33] Machado FS Dutra WO Esper L Gollob K Teixeira M Factor SM. Current understanding of immunity to Trypanosoma cruzi infection and pathogenesis os Chagas disease. Semin Immunopathol (2013) 34:753–70. 10.1007/s00281-012-0351-7.Current PMC3498515 23076807

[34] Sallusto F Lanzavecchia A. Efficient Presentation of Soluble Antigen by Cultured Human Dendritic Cells Is Maintained by Granulocyte/Macrophage Colony-stimulating Factor Plus Interleukin 4 and Downregulated by Tumor Necrosis Factor-alpha. J Exp Med (1994) 179:1109–18. 10.1084/jem.179.4.1109 PMC2191432 8145033

[35] Poncini CV González-Cappa SM. Dual role of monocyte-derived dendritic cells in Trypanosoma cruzi infection. Eur J Immunol (2017) 47:1936–48. 10.1002/eji.201646830 28748529

[36] Banchereau J Briere F Caux C Davoust J Lebecque S Liu Y. Immunobiology of dendritic cells. Annu Rev Immunol (2000) 18:767–811. 10.1146/annurev.immunol.18.1.767 10837075

[37] Amigorena S. Dendritic Cells on the Way to Glory. J Immunol (2018) 200:885–6. 10.4049/jimmunol.1701693 29358411

[38] Brodskyn C Patricio J Oliveira R Lobo L Arnholdt A Mendonça-previato L. Glycoinositolphospholipids from Trypanosoma cruzi Interfere with Macrophages and Dendritic Cell Responses. Infect Immun (2002) 70:3736–43. 10.1128/IAI.70.7.3736-3743.2002 PMC128086 12065516

[39] Van Overtvelt L Vanderheyde N Verhasselt V Ismaili J De Vos L Goldman M. Trypanosoma cruzi infects human dendritic cells and prevents their maturation: inhibition of cytokines, HLA-DR, and costimulatory molecules. Infect Immun (1999) 67:4033–40. 10.1128/IAI.67.8.4033-4040.1999 PMC96695 10417171

[40] Van Overtvelt L Andrieu M Verhasselt V Connan F Choppin J Vercruysse V. Trypanosoma cruzi down-regulates lipopolysaccharide-induced MHC class I on human dendritic cells and impairs antigen presentation to specific CD8 + T lymphocytes. Int Immunol (2002) 14:1135–44. 10.1093/intimm/dxf077 12356679

[41] Mendes MT Carvalho-Costa TM Da Silva MV Anhê ACBM Guimarães RM Da Costa TA. Effect of the saliva from different triatomine species on the biology and immunity of TLR-4 ligand and Trypanosoma cruzi-stimulated dendritic cells. Parasites Vectors (2016) 9:634. 10.1186/s13071-016-1890-x 27938380 PMC5148907

[42] Barbosa CG Carvalho Costa TM Desidério CS Ferreira PTM Silva MDO Hernández CG. Trypanosoma cruzi mexican strains differentially modulate surface markers and cytokine production in bone marrow-derived dendritic cells from C57BL/6 and BALB/c Mice. Mediators Inflamm (2019) 2019:7214798. 10.1155/2019/7214798 31636507 PMC6766131

[43] da Costa TA Silva MV Mendes MT Carvalho-Costa TM Batista LR Lages-Silva E. Immunomodulation by *Trypanosoma cruzi* : Toward Understanding the Association of Dendritic Cells with Infecting TcI and TcII Populations. J Immunol Res (2014) 2014:1–12. 10.1155/2014/962047 PMC4211313 25371910

[44] Planelles L Thomas MC Marañón C Morell M López MC. Differential CD86 and CD40 co-stimulatory molecules and cytokine expression pattern induced by Trypanosoma cruzi in APCs from resistant or susceptible mice. Clin Exp Immunol (2003) 131:41–7. 10.1046/j.1365-2249.2003.02022.x PMC1808596 12519384

[45] Gil-Jaramillo N Motta FN Favali CBF Bastos IM Santana JM. Dendritic cells: a double-edged sword in immune responses during Chagas Disease. Front Microbiol (2016) 7:1076. 10.3389/fmicb.2016.01076 27471496 PMC4943928

[46] Camargo EP. Growth and differentiation in Trypanosoma cruzi. Origin of metacyclic trypanosomes in liquid media. Rev do Inst Med Trop São Paulo (1964) 6:93–100. 14177814

[47] Contreras VT Salles JM Thomas N Morel CM Goldenberg S. In vitro differentiation of Trypanosoma cruzi under chemically defined conditions. Mol Biochem Parasitol (1985) 16:315–27. 10.1016/0166-6851(85)90073-8 3903496

[48] Canavaci AMC Bustamante JM Padilla AM Perez Brandan CM Simpson LJ Xu D. In vitro and in vivo high-throughput assays for the testing of anti-Trypanosoma cruzi compounds. PLoS Negl Trop Dis (2010) 4:e740. 10.1371/journal.pntd.0000740 20644616 PMC2903469

[49] Moser DR Kirchhoff LV Donelson JE. Detection of Trypanosoma cruzi by DNA amplification using the polymerase chain reaction. J Clin Microbiol (1989) 27:1477–82. 10.1128/JCM.27.7.1477-1482.1989 PMC267598 2504769

[50] Ebstein F Lange N Urban S Seifert U Krüger E Kloetzel PM. Maturation of human dendritic cells is accompanied by functional remodelling of the ubiquitin-proteasome system. Int J Biochem Cell Biol (2009) 41:1205–15. 10.1016/j.biocel.2008.10.023 19028597

[51] Pereira SR Faça VM Gomes GG Chammas R Fontes AM Covas DT. Changes in the proteomic profile during differentiation and maturation of human monocyte-derived dendritic cells stimulated with granulocyte macrophage colony stimulating factor/interleukin-4 and lipopolysaccharide. Proteomics (2005) 5:1186–98. 10.1002/pmic.200400988 15800872

[52] Kiama SG Cochand L Karlsson L Nicod LP Gehr P. Evaluation of phagocytic activity in human monocyte-derived dendritic cells. J Aerosol Med (2001) 14:289–99. 10.1089/089426801316970240 11693840

[53] Kim D Langmead B Salzberg SL. HISAT: A fast spliced aligner with low memory requirements. Nat Methods (2015) 12:357–60. 10.1038/nmeth.3317 PMC4655817 25751142

[54] Li H Handsaker B Wysoker A Fennell T Ruan J Homer N. The Sequence Alignment/Map format and SAMtools. Bioinformatics (2009) 25:2078–9. 10.1093/bioinformatics/btp352 PMC2723002 19505943

[55] Anders S Pyl PT Huber W. HTSeq – A Python framework to work with high-throughput sequencing data HTSeq – A Python framework to work with high-throughput sequencing data. Bioinformatics (2014) 31:0–5. 10.1093/bioinformatics/btu638 PMC4287950 25260700

[56] Love MI Huber W Anders S. Moderated estimation of fold change and dispersion for RNA-seq data with DESeq2. Genome Biol (2014) 15. 10.1186/s13059-014-0550-8 PMC4302049 25516281

[57] Beißbarth T Speed TP. GOstat: Find statistically overrepresented Gene Ontologies with a group of genes. Bioinformatics (2004) 20:1464–5. 10.1093/bioinformatics/bth088 14962934

[58] Luo W Brouwer C. Pathview: An R/Bioconductor package for pathway-based data integration and visualization. Bioinformatics (2013) 29:1830–1. 10.1093/bioinformatics/btt285 PMC3702256 23740750

[59] Szklarczyk D Morris JH Cook H Kuhn M Wyder S Simonovic M. The STRING database in 2017: Quality-controlled protein-protein association networks, made broadly accessible. Nucleic Acids Res (2017) 45:D362–8. 10.1093/nar/gkw937 PMC5210637 27924014

[60] Shannon P Markiel A Ozier O Baliga NS Wang JT Ramage D. Cytoscape: A Software Environment for Integrated Models of Biomolecular Interaction Networks. Genome Res (2003) 13:2498–504. 10.1101/gr.1239303 PMC403769 14597658

[61] Plaisier CL Horvath S Huertas-Vazquez A Cruz-Bautista I Herrera MF Tusie-Luna T. Pajukanta P. A Systems Genetics Approach Implicates USF1, FADS3, and Other Causal Candidate Genes for Familial Combined Hyperlipidemia. PLoS Genet (2009) 5:e1000642. 10.1371/journal.pgen.1000642 19750004 PMC2730565

[62] Livak KJ Schmittgen TD. Analysis of relative gene expression data using real-time quantitative PCR and the 2-ΔΔCT method. Methods (2001) 25:402–8. 10.1006/meth.2001.1262 11846609

[63] Willyard C. New human gene tally reignites debate. Nature (2018) 558:354–5. 10.1038/d41586-018-05462-w 29921859

[64] Covarrubias C Cortez M Ferreira D Yoshida N. Interaction with host factors exacerbates Trypanosoma cruzi cell invasion capacity upon oral infection. Int J Parasitol (2007) 37:1609–16. 10.1016/j.ijpara.2007.05.013 17640647

[65] Mosca W Briceño L. Proliferation and bystander suppression induced by membrane and flagellar antigens of Trypanosoma cruzi. Invest Clin (2009) 50:77–87. 19418729

[66] De Bona E Lidani KCF Bavia L Omidian Z Gremski LH Sandri TL. Autoimmunity in Chronic Chagas Disease: A Road of Multiple Pathways to Cardiomyopathy? Front Immunol (2018) 9:1842. 10.3389/fimmu.2018.01842 30127792 PMC6088212

[67] Rodriguez P Carlier Y Truyens C. Trypanosoma cruzi activates cord blood myeloid dendritic cells independently of cell infection. Med Microbiol Immunol (2012) 201:287–96. 10.1007/s00430-012-0230-9 22327272

[68] Magalhães LMD Viana A Chiari E Galvão LMC Gollob KJ Dutra WO. Differential Activation of Human Monocytes and Lymphocytes by Distinct Strains of Trypanosoma cruzi. PLoS Negl Trop Dis (2015) 9:e0003816. 10.1371/journal.pntd.0003816 26147698 PMC4492932

[69] Schoggins JW Wilson SJ Panis M Murphy MY Jones CT Bieniasz P. A diverse array of gene products are effectors of type I Interferon Antiviral Response. Nature (2011) 472:481–5. 10.1038/nature09907 PMC3409588 21478870

[70] Ivashkiv LB Donlin LT. Regulation of type i interferon responses. Nat Rev Immunol (2014) 14:36–49. 10.1038/nri3581 24362405 PMC4084561

[71] Li Y Shah-Simpson S Okrah K Belew AT Choi J Caradonna KL. Transcriptome Remodeling in Trypanosoma cruzi and Human Cells during Intracellular Infection. PLoS Pathog (2016) 12:e1005511. 10.1371/journal.ppat.1005511 27046031 PMC4821583

[72] Houston-Ludlam GA Belew AT El-Sayed NM. Comparative transcriptome profiling of human foreskin fibroblasts infected with the Sylvio and y strains of Trypanosoma cruzi. PLoS One (2016) 11:1–15. 10.1371/journal.pone.0159197 PMC4978399 27505626

[73] Fernandes MC Dillon LAL Belew AT Corrada Bravo H Mosser DM El-Sayed NM. Dual Transcriptome Profiling of Leishmania-infected human macrophages reveals distinct reprogramming signatures. MBio (2016) 7:1–16. 10.1128/mBio.00027-16 PMC4959658 27165796

[74] Alsharifi M Müllbacher A Regner M. Interferon type I responses in primary and secondary infections. Immunol Cell Biol (2008) 86:239–45. 10.1038/sj.icb.7100159 18180794

[75] Nguyen KB Cousens LP Doughty LA Pien GC Durbin JE Biron CA. Interferon α/β-mediated inhibition and promotion of interferon γ: STAT1 resolves a paradox. Nat Immunol (2000) 1:70–6. 10.1038/76940 10881178

[76] MacMicking J Xie QW Nathan C. Nitric oxide and macrophage function. Annu Rev Immunol (1997) 15:323–50. 10.1146/annurev.immunol.15.1.323 9143691

[77] Luft T Pang KC Thomas E Hertzog P Hart DN Trapani J. Type I IFNs enhance the terminal differentiation of dendritic cells. J Immunol (1998) 161:1947–53. 10.4049/jimmunol.178.12.7540 9712065

[78] Une C Andersson J Örn A. Role of IFN-α/β and IL-12 in the activation of natural killer cells and interferon-γ production during experimental infection with Trypanosoma cruzi. Clin Exp Immunol C Andersson J Örn A (2003) Role IFN-α/β IL-12 Act Nat Kill Cells Interf Prod Dur Exp Infect Trypanos cruzi Clin Exp I (2003) 134:195–201. 10.1046/j.1365-2249.2003.02294.x PMC1808850 14616777

[79] Costa VMA Torres KCL Mendonca RZ Gresser I Gollob KJ Abrahamsohn IA. Type I IFNs Stimulate Nitric Oxide Production and Resistance to Trypanosoma cruzi Infection. J Immunol (2006) 177:3193–200. 10.4049/jimmunol.177.5.3193 16920958

[80] Koga R Hamano S Kuwata H Atarashi K Ogawa M Hisaeda H. TLR-Dependent Induction of IFN- β Mediates Host Defense against Trypanosoma cruzi. J Immunol (2006) 177:7059–66. 10.4049/jimmunol.177.10.7059 17082622

[81] Chessler A-DC Unnikrishnan M Bei AK Daily JP Burleigh BA. Trypanosoma cruzi Triggers an Early Type I IFN Response In Vivo at the Site of Intradermal Infection. J Immunol (2009) 182:2288–96. 10.4049/jimmunol.0800621 19201883

[82] Martinon F Mayor A Tschopp J. The Inflammasomes: Guardians of the Body. Annu Rev Immunol (2009) 27:229–65. 10.1146/annurev.immunol.021908.132715 19302040

[83] Gonçalves VM Matteucci KC Buzzo CL Miollo BH Ferrante D Torrecilhas AC. NLRP3 Controls Trypanosoma cruzi Infection through a Caspase-1-Dependent IL-1R-Independent NO Production. PLoS Negl Trop Dis (2013) 7:e2469. 10.1371/journal.pntd.0002469 24098823 PMC3789781

[84] Silva GK Costa RS Silveira TN Caetano BC Horta CV Gutierrez FRS. Apoptosis-Associated Speck–like Protein Containing a Caspase Recruitment Domain Inflammasomes Mediate IL-1β Response and Host Resistance to Trypanosoma cruzi Infection. J Immunol (2013) 191:3373–83. 10.4049/jimmunol.1203293 23966627

[85] Dey N Sinha M Gupta S Gonzalez MN Fang R Endsley JJ. Caspase-1/ASC inflammasome-mediated activation of IL-1β-ROS-NF-κB pathway for control of Trypanosoma cruzi replication and survival is dispensable in NLRP3-/- macrophages. PLoS One (2014) 9:e111539. 10.1371/journal.pone.0111539 25372293 PMC4221042

[86] Huante MB Gupta S Calderon VC Koo SJ Sinha M Luxon BA. Differential inflammasome activation signatures following intracellular infection of human macrophages with Mycobacterium bovis BCG or Trypanosoma cruzi. Tuberculosis (2016) 101:S35–44. 10.1016/j.tube.2016.09.026 PMC7418480 27733245

[87] Poeck H Bscheider M Gross O Finger K Roth S Rebsamen M. Recognition of RNA virus by RIG-I results in activation of CARD9 and inflammasome signaling for interleukin 1β production. Nat Immunol (2010) 11:63–9. 10.1038/ni.1824 19915568

[88] Fernandes-Alnemri T Yu JW Datta P Wu J Alnemri ES. AIM2 activates the inflammasome and cell death in response to cytoplasmic DNA. Nature (2009) 458:509–13. 10.1038/nature07710 PMC2862225 19158676

[89] Kerur N Veettil MV Sharma-Walia N Bottero V Sadagopan S Otageri P. IFI16 acts as a nuclear pathogen sensor to induce the inflammasome in response to Kaposi Sarcoma-associated herpesvirus infection. Cell Host Microbe (2011) 9:363–75. 10.1016/j.chom.2011.04.008 PMC3113467 21575908

[90] Karki R Man SM Malireddi RKS Gurung P Vogel P Lamkanfi M. Concerted activation of the AIM2 and NLRP3 inflammasomes orchestrates host protection against aspergillus infection. Cell Host Microbe (2015) 17:657–368. 10.1016/j.chom.2015.01.006 PMC4359672 25704009

[91] Kalantari P DeOliveira RB Chan J Corbett Y Rathinam V Stutz A. Dual engagement of the NLRP3 and AIM2 inflammasomes by plasmodium-derived hemozoin and DNA during Malaria. Cell Rep (2014) 6:196–210. 10.1016/j.celrep.2013.12.014 24388751 PMC4105362

[92] Zamboni DS Lima-Junior DS. Inflammasomes in host response to protozoan parasites. Immunol Rev (2015) 265:156–71. 10.1111/imr.12291 25879291

[93] van de Weijer ML Luteijn RD Wiertz EJHJ. Viral immune evasion: Lessons in MHC class I antigen presentation. Semin Immunol (2015) 27:125–37. 10.1016/j.smim.2015.03.010 25887630

[94] Vignesh KS Deepe GS. Metallothioneins: Emerging modulators in immunity and infection. Int J Mol Sci (2017) 18:2197. 10.3390/ijms18102197 PMC5666878 29065550

[95] Molteni CG Principi N Esposito S. Reactive oxygen and nitrogen species during viral infections. Free Radic Res (2014) 48:1163–9. 10.3109/10715762.2014.945443 25039433

[96] Kim T-S Shin E-C. The activation of bystander CD8 + T cells and their roles in viral infection. Exp Mol Med (2019) 51:154. 10.1038/s12276-019-0316-1 PMC6906361 31827070

[97] Sant AJ McMichael A. Revealing the role of CD4+ T cells in viral immunity. J Exp Med (2012) 209:1391–5. 10.1084/jem.20121517 PMC3420330 22851641

[98] Basso B. Modulation of immune response in experimental Chagas disease. World J Exp Med (2013) 3:1–10. 10.5493/wjem.v3.i1.World 24520540 PMC3905588

[99] Blanco P Palucka AK Pascual V Banchereau J. Dendritic cells and cytokines in human inflammatory and autoimmune diseases. Cytokine Growth Factor Rev (2009) 19:41–52. 10.1016/j.cytogfr.2007.10.004 PMC2413068 18258476

[100] Silva JS Vespa GNR Cardoso MAG Aliberti JCS Cunha FQ. Tumor necrosis factor alpha mediates resistance to Trypanosoma cruzi infection in mice by inducing nitric oxide production in infected gamma interferon-activated macrophages. Infect Immun (1995) 63:4862–7. 10.1128/iai.63.12.4862-4867.1995 PMC173696 7591147

[101] Guilmot A Bosse J Carlier Y Truyens C. Monocytes Play an IL-12-Dependent Crucial Role in Driving Cord Blood NK Cells to Produce IFN-g in Response to Trypanosoma cruzi. PLoS Negl Trop Dis (2013) 7:e2291. 10.1371/journal.pntd.0002291 23819002 PMC3688561

[102] Poncini CV Soto CDA Batalla E Solana ME Gonzalez Cappa SM. Trypanosoma cruzi Induces Regulatory Dendritic Cells In Vitro. Infect Immun (2008) 76:2633–41. 10.1128/IAI.01298-07 PMC2423106 18347042

[103] Lee SH Stehlik C Reed JC. COP, a Caspase Recruitment Domain-containing Protein and Inhibitor of Caspase-1 Activation Processing. J Biol Chem (2001) 276:34495–500. 10.1074/jbc.M101415200 11432859

[104] Druilhe A Srinivasula SM Razmara M Ahmad M Alnemri ES. Regulation of IL-1β generation by Pseudo-ICE and ICEBERG, two dominant negative caspase recruitment domain proteins. Cell Death Differ (2001) 8:649–57. 10.1038/sj.cdd.4400881 11536016

[105] Chang EY Guo B Doyle SE Cheng G. Cutting Edge: Involvement of the Type I IFN Production and Signaling Pathway in Lipopolysaccharide-Induced IL-10 Production. J Immunol (2007) 178:6705–9. 10.4049/jimmunol.178.11.6705 17513714

[106] Moreira LO El Kasmi KC Smith AM Finkelstein D Fillon S Kim YG. The TLR2-MyD88-NOD2-RIPK2 signalling axis regulates a balanced pro-inflammatory and IL-10-mediated anti-inflammatory cytokine response to Gram-positive cell walls. Cell Microbiol (2008) 10:2067–77. 10.1111/j.1462-5822.2008.01189.x PMC4966886 18549453

[107] Hovingh ES van Gent M Hamstra H-J Demkes M Mooi FR Pinelli E. Emerging Bordetella pertussis Strains Induce Enhanced Signaling of Human Pattern Recognition Receptors TLR2, NOD2 and Secretion of IL-10 by Dendritic Cells. PLoS One (2017) 12:e0170027. 10.1371/journal.pone.0170027 28076445 PMC5226795

[108] Jamontt J Petit S Clark N Parkinson SJ Smith P. Nucleotide-Binding Oligomerization Domain 2 Signaling Promotes Hyperresponsive Macrophages and Colitis in IL-10–Deficient Mice. J Immunol (2013) 190:2948–58. 10.4049/jimmunol.1201332 PMC3586975 23396949

[109] Hedl M Abraham C. Secretory Mediators Regulate Nod2-Induced Tolerance in Human Macrophages in Human Macrophages. Gastroenterology (2011) 140:231–41. 10.1053/j.gastro.2010.09.009 PMC3145247 20854823

[110] Netea MG Ferwerda G de Jong DJ Jansen T Jacobs L Kramer M. Nucleotide-Binding Oligomerization Domain-2 Modulates Specific TLR Pathways for the Induction of Cytokine Release. J Immunol (2005) 174:6518–23. 10.4049/jimmunol.174.10.6518 15879155

[111] Powell MJ Thompson SAJ Tone Y Waldmann H Tone M. Posttranscriptional Regulation of IL-10 Gene Expression Through Sequences in the 3′-Untranslated Region. J Immunol (2000) 165:292–6. 10.4049/jimmunol.165.1.292 10861064

[112] Brown CY Lagnado CA Vadas MA Goodall GJ. Differential regulation of the stability of cytokine mRNAs in lipopolysaccharide-activated blood monocytes in response to interleukin-10. J Biol Chem (1996) 271:20108–12. 10.1074/jbc.271.33.20108 8702732

[113] Kishore R Tebo JM Kolosov M Hamilton TA. Cutting Edge: Clustered AU-Rich Elements Are the Target of IL-10-Mediated mRNA Destabilization in Mouse Macrophages. J Immunol (1999) 162:2457–61. 10072482

[114] Block BA Arts RJW van Crevel R Benn CS Netea MG. Trained innate immunity as underlying mechanism for the long-term, nonspecific effects of vaccines. J Leukoc Biol (2015) 98:347–56. 10.1189/jlb.5ri0315-096r 26150551

[115] van Crevel R Netea MG Koeken VACM Joosten LAB Benn CS de Bree LCJ. Non-specific effects of vaccines: Current evidence and potential implications. Semin Immunol (2018) 39:35–43. 10.1016/j.smim.2018.06.002 30007489

[116] Tokunaga R Zhang W Naseem M Puccini A Berger MD Soni S. CXCL9, CXCL10, CXCL11/CXCR3 axis for immune activation – A target for novel cancer therapy. Cancer Treat Rev (2018) 63:40–7. 10.1016/j.ctrv.2017.11.007 PMC5801162 29207310

[117] Croft M Siegel RM. Beyond TNF: TNF superfamily cytokines as targets for the treatment of rheumatic diseases. Nat Rev Rheumatol (2017) 13:217–33. 10.1038/nrrheum.2017.22 PMC5486401 28275260

[118] Cong X-L Lo M-C Reuter BA Yan M Fan J-B Zhang D-E. Usp18 promotes conventional CD11b+ dendritic cell development. J Immunol (2012) 188:4776–81. 10.4049/jimmunol.1101609 PMC3345079 22491252

[119] Konermann C Kresse A Beuter-Gunia C Würthner J Degrandi D Pfeffer K. In silico and in vitro characterization of mGBP4 splice variants. DNA Cell Biol (2007) 26:847–51. 10.1089/dna.2007.0637 17919070

[120] Hu Y Wang J Yang B Zheng N Qin M Ji Y. Guanylate Binding Protein 4 Negatively Regulates Virus-Induced Type I IFN and Antiviral Response by Targeting IFN Regulatory Factor 7. J Immunol (2011) 187:6456–62. 10.4049/jimmunol.1003691 22095711

[121] Tyrkalska SD Candel S Angosto D Gómez-Abellán V Martín-Sánchez F García-Moreno D. Neutrophils mediate Salmonella Typhimurium clearance through the GBP4 inflammasome-dependent production of prostaglandins. Nat Commun (2016) 7:1–17. 10.1038/ncomms12077 PMC4932187 27363812

[122] Haller O Staeheli P Schwemmle M Kochs G. Mx GTPases: Dynamin-like antiviral machines of innate immunity. Trends Microbiol (2015) 23:154–63. 10.1016/j.tim.2014.12.003 25572883

[123] Pillai PS Molony RD Martinod K Dong H Pang IK Tal MC. Mx1 reveals innate pathways to antiviral resistance and lethal influenza disease. Science (80- ) (2016) 352:463–6. 10.1126/science.aaf3926 PMC5465864 27102485

[124] Ghosh A Shao L Sampath P Zhao B Patel NV Zhu J. Oligoadenylate-Synthetase-Family Protein OASL Inhibits Activity of the DNA Sensor cGAS during DNA Virus Infection to Limit Interferon Production. Immunity (2019) 50:51–63.e5. 10.1016/j.immuni.2018.12.013 30635239 PMC6342484

[125] Choi UY Kang JS Hwang YS Kim YJ. Oligoadenylate synthase-like (OASL) proteins: dual functions and associations with diseases. Exp Mol Med (2015) 47:e144. 10.1038/emm.2014.110 25744296 PMC4351405

[126] Gatica-Andrades M Vagenas D Kling J Nguyen TTK Benham H Thomas R. WNT ligands contribute to the immune response during septic shock and amplify endotoxemia-driven inflammation in mice. Blood Adv (2017) 1:1274–86. 10.1182/bloodadvances.2017006163 PMC5728549 29296769

[127] de Rezende MM Ng-Blichfeldt JP Justo GZ Paredes-Gamero EJ Gosens R. Divergent effects of Wnt5b on IL-3- and GM-CSF-induced myeloid differentiation. Cell Signal (2020) 67:109507. 10.1016/j.cellsig.2019.109507 31857239 PMC7116107

[128] Wang L Zhou Y Chen Z Sun L Wu J Li H. PLCβ2 negatively regulates the inflammatory response to virus infection by inhibiting phosphoinositide-mediated activation of TAK1. Nat Commun (2019) 10:1–13. 10.1038/s41467-019-08524-3 30765691 PMC6375925

[129] Dolganiuc A Kodys K Kopasz A Marshall C Do T Romics L. Hepatitis C Virus Core and Nonstructural Protein 3 Proteins Induce Pro- and Anti-inflammatory Cytokines and Inhibit Dendritic Cell Differentiation. J Immunol (2003) 170:5615–24. 10.4049/jimmunol.170.11.5615 12759441

[130] Yonejima A Mizukoshi E Tamai T Nakagawa H Kitahara M Yamashita T. Characteristics of Impaired Dendritic Cell Function in Patients With Hepatitis B Virus Infection. Hepatology (2019) 70:25–39. 10.1002/hep.30637/suppinfo 30938456

[131] Roberts LL Robinson CM. Mycobacterium tuberculosis infection of human dendritic cells decreases integrin expression, adhesion and migration to chemokines. Immunology (2013) 141:39–51. 10.1111/imm.12164 PMC3893848 23981064

[132] Urban BC Ferguson DJ Pain A Willcox N Plebanski M Austyn JM. Plasmodium falciparum infected erythrocytes modulate the maturation of dendritic cells. Nature (1999) 400:73–7. 10.1038/21900 10403251

