# Supplementary material for: The First Contact of Human Dendritic Cells With Trypanosoma cruzi Reveals Response to Virus as an Unexplored Central Pathway
Source: Front Immunol. 2021 Apr 9;12:638020. doi: 10.3389/fimmu.2021.638020 (PMC8062726; doi:10.3389/fimmu.2021.638020)
Supplement: Supplementary file 1 [file DataSheet_1.docx]

Supplementary Material

# Supplementary Figures


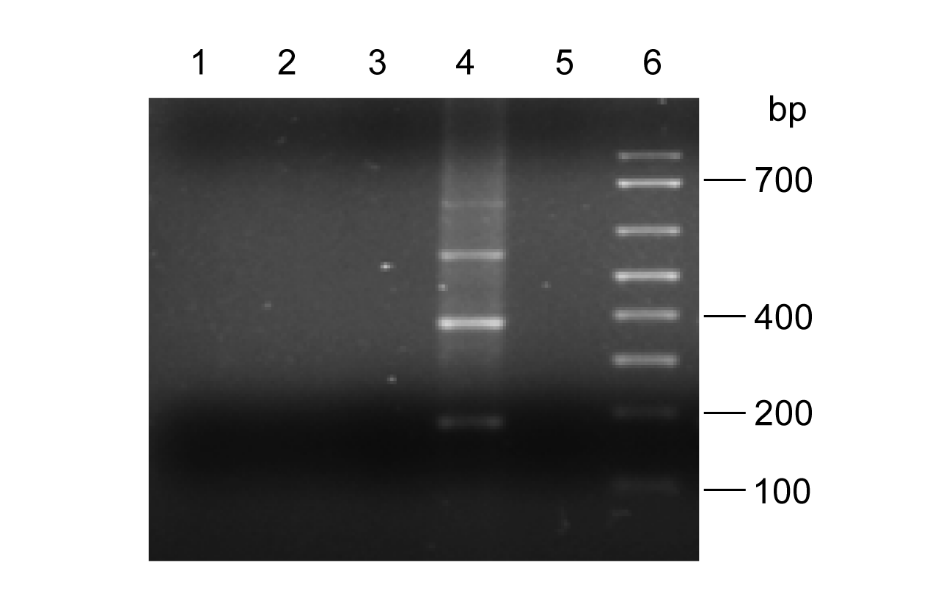


**Supplementary Figure 1.** **TCZ PCR to confirm no *T. cruzi* infection in the donors** (Related to Figure 1). TCZ region (188 bp) in the *T. cruzi* genome was amplified from total DNA extracted from donors' PBMCs. PCR products from donor A (line 1), donor B (line 2), donor C (line 3), *T. cruzi* total DNA (line 4), and non-infected donor (line5) are shown in a 1% Agarose gel.


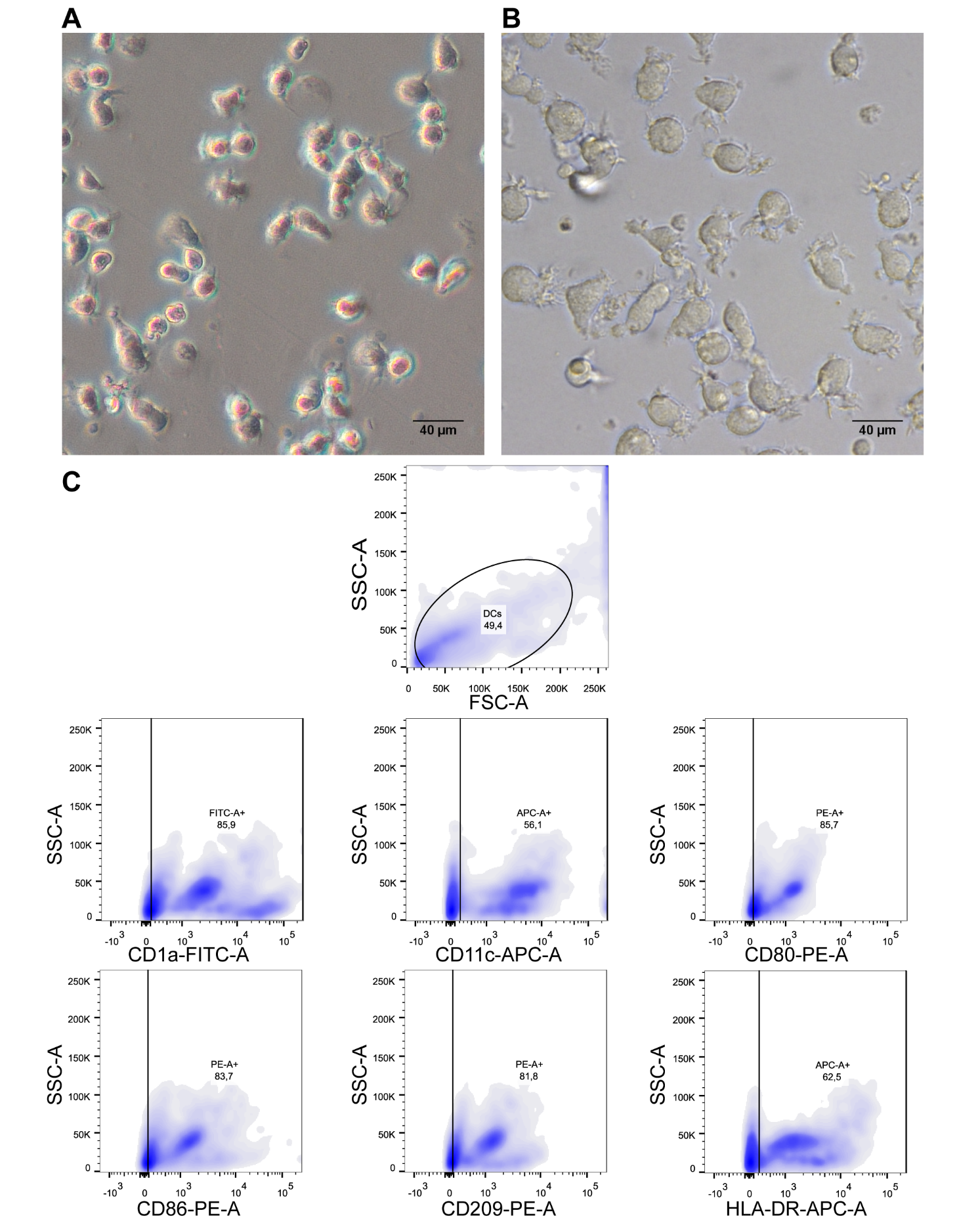


**Supplementary Figure 2. Human dendritic cells differentiation** (Related to Figure 1). Monocytes were purified from peripheral blood of donors (A) and differentiated into MoDCs in the presence of IL-4 and GM-CSF cytokines during 7 days (B) (Scale bar: 40 µm). Flow cytometry was performed to assess the differentiation efficiency, evaluating expression of CD1a, CD11c, CD80, CD86, CD209, and HLA-DR. The upper density plot shows the whole population and the selected dendritic cell gate. The other density plots show the positive populations within the selected gate (C). Data from a representative experiment.


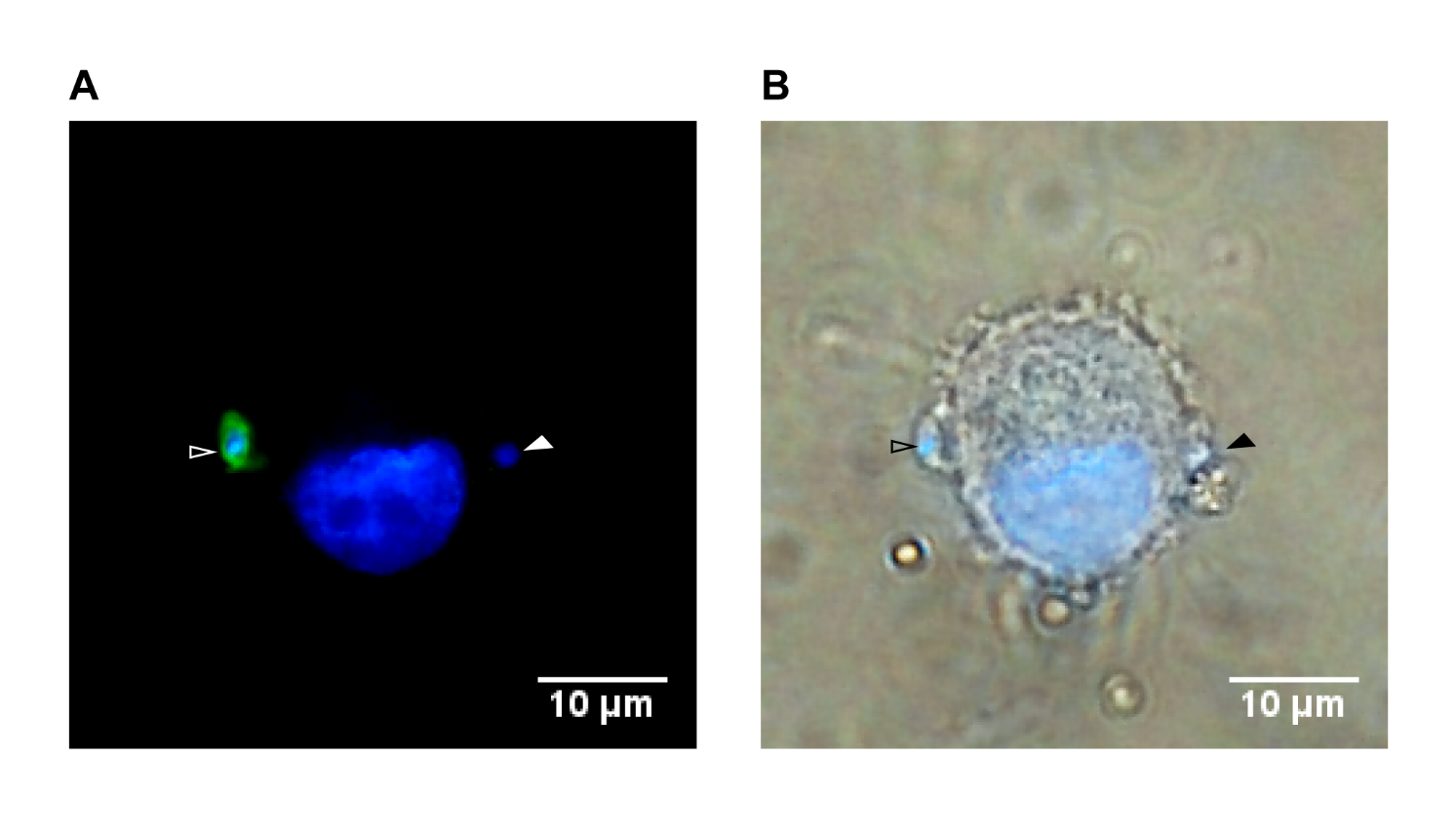


**Supplementary Figure 3. Percentage of infected cells in the Infected group** (Related to Figure 1). To identify intracellular amastigotes, in/out immunofluorescence was performed using chagasic mice serum. MoDCs were not permeabilized in order to recognize extracellular parasites (green). DAPI was used to detect nuclei (blue). A representative fluorescence micrograph is shown in A, and its respective phase-contrast micrograph is shown in B. A full arrow shows an intracellular amastigote inside the MoDC, whereas an empty arrow shows an extracellular parasite. Scale bar: 10 µm.

# Supplementary Table Legends

**Supplementary Table 1. Immunizations of donors** (Related to Figure 1). Viral infection and vaccination data from each donor.

**Supplementary Table 2. Mapping results** (Related to Figure 2). Samples, raw reads, number of mapped reads simultaneously in human and *T. cruzi* genomes, and corresponding percentages by sample. A, B, and C: donors; 1 and 2: replicate number; C: control condition, I: infected condition, and donors.

**Supplementary Table 3. Percentage of genes mapped in each genome** (Related to Figure 2). Read counts and percentage mapped in each genome.

**Supplementary Table 4. Differential expression analysis** (Related to Figure 2). DEGs detected in human MoDCs after 12 h of infection with *T. cruzi* represented by gene IDs, gene symbols and gene complete names. The data also includes Log_2_ fold change, adjusted p-value, the average of the normalized counts considering all samples (baseMean), Log fold change standard error (ifcSE), Wald statistic (stat), p-value and entrez number for each gene.

**Supplementary Table 5. Enriched Gene Ontology terms** (Related to Figure 3). Gene Ontology (GO) enrichment analysis represented by number ID of gene ontology biological process (GOBPID), p-value, odds ratio, expected count, observed count, and total number of genes from a GO term.

**Supplementary Table 6. Enriched KEGG pathways** (Related to Figure 3). KEGG enrichment analysis represented by ID, description of pathway, frequency of DEGs compared to total genes from the pathway (GeneRatio), frequency of genes annotated from the pathway compared to total genes annotated from background (BgRatio), p-value, adjusted p-value, q-value, gene ID and number of DEGs within the pathway.
